# Effect of Au substrate and coating on the lasing characteristics of GaAs nanowires

**DOI:** 10.1038/s41598-021-00855-w

**Published:** 2021-11-01

**Authors:** Gyanan Aman, Fatemesadat Mohammadi, Martin Fränzl, Mykhaylo Lysevych, Hark Hoe Tan, Chennupati Jagadish, Heidrun Schmitzer, Marc Cahay, Hans Peter Wagner

**Affiliations:** 1 Department of Electrical Engineering and Computer Science, University of Cincinnati, Cincinnati, OH 45221 USA; 2 Department of Physics, University of Cincinnati, Cincinnati, OH 45221 USA; 3 Department of Physics, University of Leipzig, 04109 Leipzig, Germany; 4 Department of Electronic Materials Engineering, ARC Center of Excellence for Transformative Meta-Optical Systems, Research School of Physics, The Australian National University, Canberra, ACT 2601 Australia; 5 Department of Physics, Xavier University, Cincinnati, OH 45207 USA

**Keywords:** Materials science, Nanoscience and technology, Optics and photonics

## Abstract

Optically pumped lasing from highly Zn-doped GaAs nanowires lying on an Au film substrate and from Au-coated nanowires has been demonstrated up to room temperature. The conically shaped GaAs nanowires were first coated with a 5 nm thick Al_2_O_3_ shell to suppress atmospheric oxidation and band-bending effects. Doping with a high Zn concentration increases both the radiative efficiency and the material gain and leads to lasing up to room temperature. A detailed analysis of the observed lasing behavior, using finite-difference time domain simulations, reveals that the lasing occurs from low loss hybrid modes with predominately photonic character combined with electric field enhancement effects. Achieving low loss lasing from NWs on an Au film and from Au coated nanowires opens new prospects for on-chip integration of nanolasers with new functionalities including electro-optical modulation, conductive shielding, and polarization control.

## Introduction

Nanophotonics aims to diminish the size mismatch between electronics and photonics to enable optical on-chip applications including coherent light generation and optical data transfer at the nanometer scale. The emerging optoelectronic integrated circuits (OEIC) offer significantly enhanced bandwidth as well as reduced power consumption per generated bit^1,2^. Amongst the promising photonic material toward the realization of OEICs are semiconductors nanowires (NWs)^3,4^ and nanowire arrays^5,6^ which have led to nanophotonic device demonstrations including waveguides^7–9^, light emitting diodes^10–12^, single NW laser^6,13–23^ and photonic crystal NW laser arrays^24–28^. Different III–V and II–VI semiconductor materials and alloys allow the tuning of laser emission from the ultraviolet to the infrared spectral region. The diameter of single NW waveguides and laser cavities is fundamentally constrained by the diffraction limit of the guided photonic modes^20,29–31^. Optical waveguiding and lasing below the optical diffraction limit can be achieved by placing the semiconductor nanowires on a noble metal substrate. The resulting plasmonic mode is concentrated near the metal/semiconductor interface in an ultra-small mode volume^32^. The high surface plasmon-polariton (SPP) losses are partially compensated by an enhanced mode confinement factor and modal gain^33^, an increased Purcell factor^34–36^, an increased spontaneous emission factor and low group velocities^37–39^. However, purely plasmonic GaAs NW lasers still require high optical pump intensities to overcome the plasmonic losses, which prevents optically pumped lasing at room temperature^39^ and OEIC applications. In order to advance the performance of hybrid Au/GaAs-based NW lasers light-matter interaction in hybrid NW systems is investigated as it combines the advantages of both low loss photonic lasing and plasmonic electric field enhancement at the NW/metal interface and in the vicinity of metal nanoparticles.

In this work, we explore the benefits of low-loss photonic-plasmonic lasing in hybrid Au/GaAs NWs, which have tip diameters ranging from 200 to 320 nm. In particular, we investigate optically pumped lasing from highly Zn-doped GaAs NWs on Au film substrate as well as from Au-coated NWs at temperatures ranging from 77 K up to room temperature. High Zn-doping in GaAs NWs significantly enhances the radiative efficiency by reducing the radiative lifetime so that the photoexcited carriers radiatively recombine before they are captured at surface traps. In addition, high Zn doping increases the differential gain and reduces the transparency carrier density due to the high hole concentration already present^14^. A thin Al_2_O_3_ spacer layer of ~ 5 nm in thickness was deposited around the NWs to suppress atmospheric oxidation and to reduce metal induced band bending^40,41^, at the Au/GaAs interface. As a new approach, we deposited a nominally 10 nm thick Au film around the NWs resulting in a coaxial hybrid Au/Al_2_O_3_/GaAs shell-core NW laser structure. This design has the advantage that it offers plasmonic electric-field (E-field) enhancement effects without the requirement of a metal substrate, which is beneficial for integrating nanolasing devices on silicon-on insulator (SOI) platforms. The lasing experiments were interpreted using finite-difference time-domain (FDTD) simulations and by calculating the output lasing power versus excitation pump power (*L–L* plot) using an extended coupled rate equation model. Our experiments demonstrate that the threshold power of a photonic NW laser can be reduced by placing the NW on a Au film because the absorption cross section of the pump E-field is plasmonically enhanced. Our investigations open new prospects for on-chip integration of nanophotonic devices with specific functionalities, including the design of electric-field modulated^42,43^, or shielded GaAs-based NW lasers as well as the development of asymmetrically (half-sided) Au coated NW laser structures with plasmonically induced chiral emission^44^. Our results strongly promote the development of electrically driven nanowire lasers^45,46^, and of metal cladded semiconductor nanolasers^47,48^. For doped GaAs NWs, the length of *p*- and *n*-segments of the *p–n* junction can be designed such that most radiative recombination occurs in the *p*-region. As an alternative, a highly doped *n*-type shell around the *p*-type GaAs core would allow recombination primarily in the *p*-GaAs core. Both laser designs have potential to provide coherent light sources for photonic integrated circuits (PIC_S_)^49^.

## Results and discussion

### Growth and structural characterization of the nanowires

**Figure 1:**
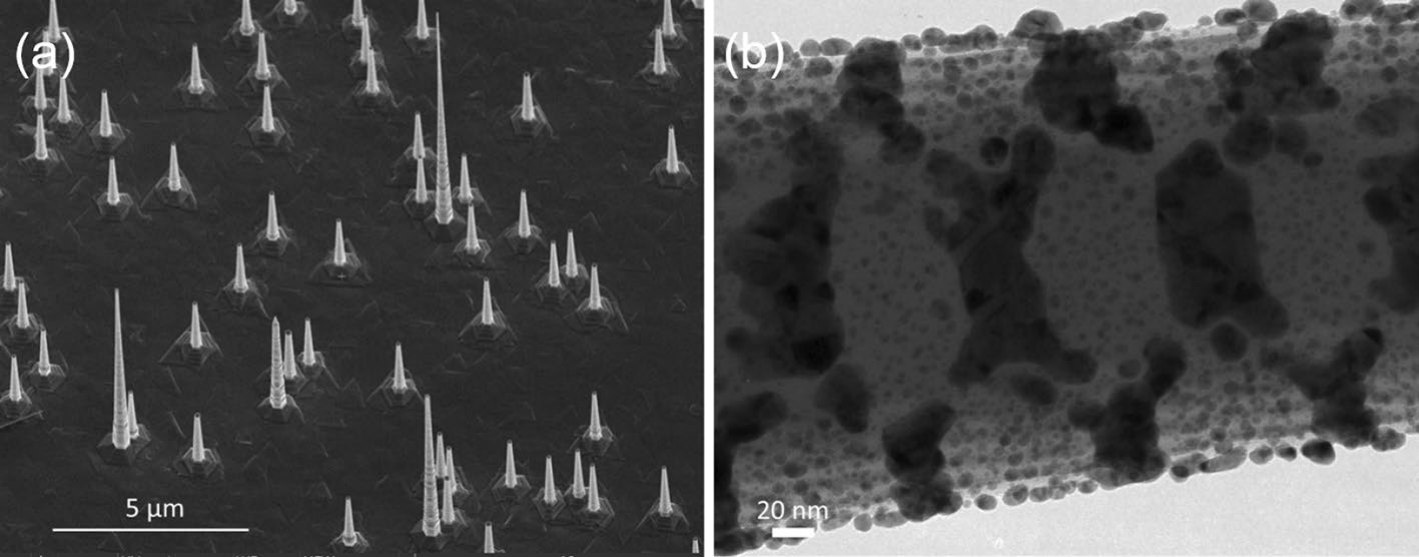
Structural characterization of GaAs NWs. (**a**) SEM image of highly Zn-doped GaAs NWs on GaAs substrate recorded at a tilt angle of 30°. The typical length of the NWs is ~3 µm with a few NWs longer than 8 µm. (**b**) TEM image of a section of a GaAs NW with a 5 nm Al_2_O_3_ coating and with a nominally 10 nm Au layer around it. The Au film is not continuous, and Au islands and nanoparticles are formed instead.

The GaAs NWs were grown by Au-catalyzed metal organic vapor phase epitaxy (MOVPE)^14^. The size of the Au catalysts was 100 nm. In this growth regime, high Zn-doping (*p* ≈ 2 × 10^19^ cm^−3^) leads to a transformation from pure wurtzite crystal structure to a zinc blende twinning superlattice (TSL) structures (see TEM image in Supplementary Fig. S1(b) in the Supplementary Information (SI)). The NWs were subsequently coated conformally with a 5 nm thick Al_2_O_3_ film using atomic layer deposition (ALD) in order to reduce atmospheric oxidation and metal induced band-bending. ^40,41^ More details about the growth and the optical characterization of these NWs can be found in Ref.^14^ and in the SI Sect. 1(a). A scanning electron microscopy (SEM) image of the Al_2_O_3_/GaAs NW on the GaAs substrate is shown in Fig. 1a. The SEM image reveals tapered NWs with a typical length of ~ 3 µm and average diameter of ~ 220 nm.

To achieve hybrid lasing without the need of a metal substrate, a nominally 10 nm-thick Au layer was deposited around the surface of the Al_2_O_3_/GaAs NW by e-beam evaporation. Figure 1b shows a TEM image of the Au coated NW revealing the Au nanoplatelets and nanoparticles are formed, rather than a continuous film as a result of different Au sticking coefficients on the alternating TSL facet surfaces. The deposited Au nanoparticles range from 5 to 25 nm in size.

The uncoated or Au-coated GaAs NWs were mechanically removed from their GaAs substrate by either sliding the NWs sample over a 200 nm-thick Au film-coated glass over a bare glass substrate (method 1) or by using a thin brush to pick off and transfer GaAs NWs to the desired substrates (method 2). A more detailed description is given in the SI Sect. 1(b). Transfer methods 1 and 2 resulted in significantly different nanowire densities on the substrates. Method 1 led to a high NW density of more than 15 NWs within the excitation laser spot of 40 µm, whereas method 2 provided a NW density of only 1 to 5 NWs within the excitation spot (see also Supplementary Fig. S2b,c in the SI). Due to high density of nanowires on samples prepared by method 1, it was impossible to identify the lasing NWs after the investigation with a SEM or optical microscope. The much lower density provided by method 2, however, allowed the unambiguous identification of the lasing NW on the substrates (see Fig. S4). The length and tip diameters of the nanowires cores on the samples were in the range between ~ 2.5 to 6 µm and ~ 200 to ~ 320 nm. More details about the statistical distribution of the broken off NW distribution are given in the SI Sect. 1(c).

### Theoretical modelling using FDTD

The lasing experiments were complemented with finite-difference time-domain (FDTD) simulations. Waveguide mode profiles, effective refractive indices *n*_*eff*_, group refractive indices *n*_*g*_, plasmonic losses α_*p*_, absorption cross sections *σ*_abs_, reflectivity losses α_R_ and confinement factors Г of bare NWs on glass and Au and for Au-coated NWs were calculated. Because of the conical shape of the GaAs nanowires, the refractive index of a waveguide mode continuously changes within the nanowires as function of the nanowire diameter *d*. The conical shape was considered by averaging the optical parameter functions from the tip to the base of the NW. Details about the Lumerical simulations and summaries of the optical parameters for distinct nanowires are given in the SI Sect. 3(a) to (g) and in tables ST4 to ST8.

Because the GaAs core tip diameter of all investigated NWs was less than 320 nm, only the first three waveguide modes needed to be considered for lasing. The electric intensity profiles $\left| E \right|^{2}$ of the first three modes (with ascending effective refractive index) of NWs on glass (as a reference), on Au and of Au-coated NWs with a GaAs core diameter of 300 nm are depicted in Supplementary Fig. S7 in the SI Sect. 3(a). For the NWs on Au film substrate, the second hybrid waveguide mode (mode 2, see Supplementary Fig. S7e in SI) has a predominantly photonic character with significantly lower plasmonic losses (below 200 cm^−1^) compared to other hybrid plasmonic modes with losses exceeding 1500 cm^−1^. Mode 2 (evolving from photonic mode HE11b, see Supplementary Fig. S7b in the SI), is therefore the lasing mode in the experiments. In the case of Au-coated NWs, simulations with a granular air/Au effective medium (EM) layer with a 33% (*p* = 1/3) Au filling factor (see Fig. 1b) were performed. The $\left| E \right|^{2}$ mode profiles of the first three modes of the EM Au-coated NW are shown in Supplementary Fig. S7g–i. While a continuous Au coating leads to very high plasmonic losses of more than 40,000 cm^−1^, the EM Au layer has acceptable losses of the order of ~ 1000 cm^−1^. The mode profiles reveal predominantly photonic character (compared with mode profiles of bare NWs on glass shown in Supplementary Fig. S7a–c in the SI). The supported lasing modes are of type HE11a/b or TE01 depending on the tip diameter of the NW, which governs the facet reflection and confinement factor and thereby dictates the supported lasing mode.

Applying the threshold condition for lasing^15^

1 $$\Gamma g_{th} = \alpha_{p} + \frac{1}{L}\ln \left( {\frac{1}{{\sqrt {R_{t} R_{b} } }}} \right) = \alpha_{p} + \alpha_{R}$$

with *R*_t_ and *R*_b_ being the reflectivity of the top and bottom facet, respectively, allowed us to calculate the threshold gain *g*_*th*_ for individual NWs on Au film substrate, Au-coated NWs and NWs on glass substrate for reference. The threshold gain values for different NWs in the three configurations are summarized in Supplementary Tables ST4 to ST6.

### Lasing from GaAs NWs on Au film and from Au-coated NWs at 77 K

**Figure 2:**
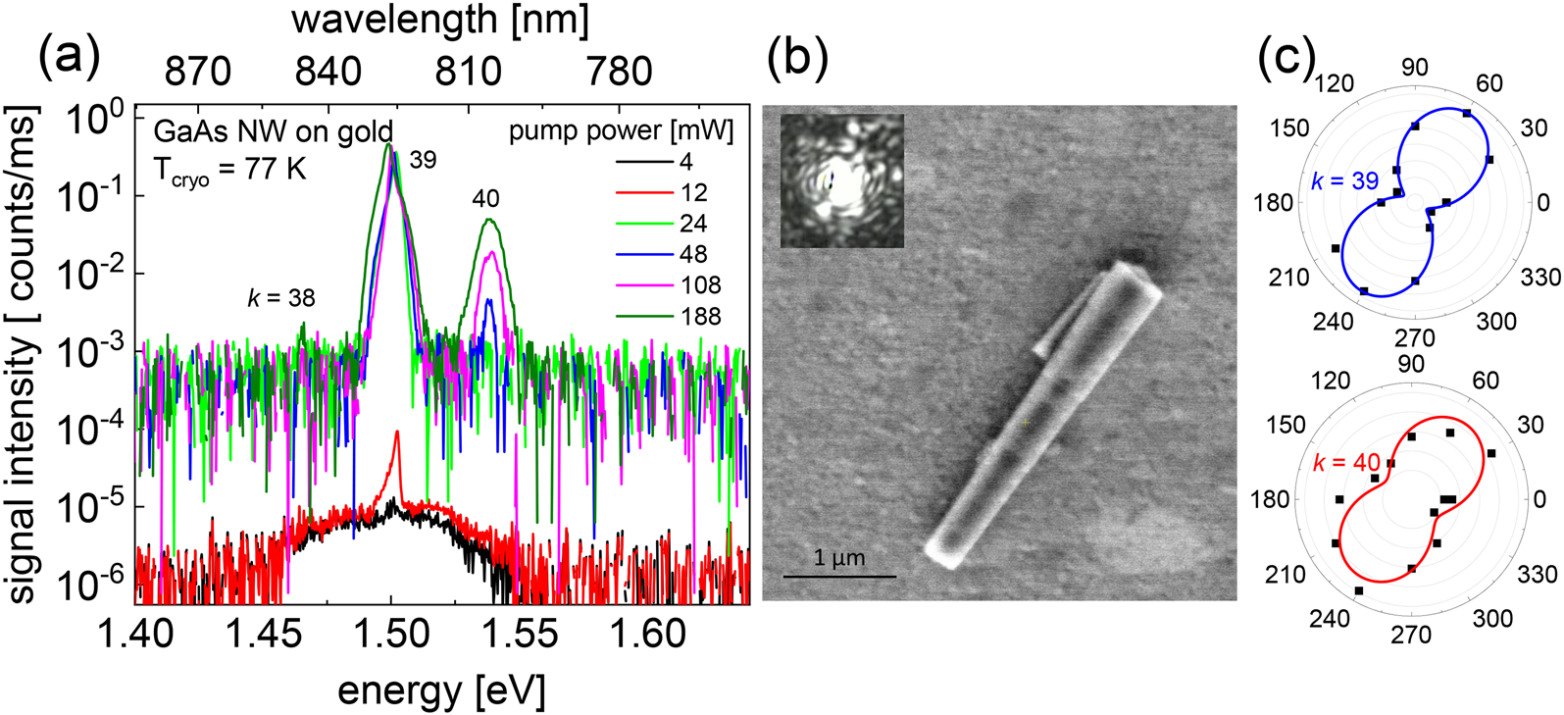
Lasing from a GaAs NW on Au film. (**a**) Pump power dependent lasing spectra of a GaAs NW on Au film at a cryostat temperature of *T*_cryo_ =77 K and excitation wavelength of λ_p_ = 720 nm. Mode numbers *k* are indicated. (**b**) SEM image of the NW investigated in (**a**). The inset shows the optical image of the NW emission at 80 mW. Interference fringes indicate coherent lasing emission. (**c**) Polar plots of longitudinal lasing modes *k* = 39 and 40.

Lasing at a cryostat temperature *T*_cryo_ = 77 K was investigated by exciting single NWs with ~ 150 fs laser pulses provided by a Ti–Sapphire laser at a repetition rate of 80 MHz with a center wavelength of 720 nm. (A detailed description of the experimental setup is given in the experimental section). Figure 2a shows excitation power dependent emission spectra of a 3.1 µm long GaAs NW on an Au film substrate, with a tip diameter of *d*_t_ = 300 nm and a base diameter of *d*_b_ = 485 nm (NW #1 in Table ST2 and ST5). The NW dimensions were obtained from the SEM image displayed in Fig. 2b. The inset in Fig. 2b shows the optical image of the NW emission at a pump power of 80 mW. The optical image reveals interference fringes indicating coherent NW laser emission. At low pump power a broad emission band appears at ~ 1.50 eV which is composed of the electron–hole recombination band ~ 1.52 eV and a lower energy emission band at ~ 1.47 eV which is attributed to Zn-related recombination^14^. Above 10 mW pump power an amplified spontaneous emission (ASE) peak emerges at 1.50 eV. With increasing pump power, a higher and lower energy longitudinal lasing mode emerges because of the increasing spectral width of the material gain (see Supplementary Fig. S18a in the SI Sect. 7). The emission from the two longitudinal modes shows the same polarization dependence when the analyzer in front of the spectrometer was rotated, shown in the polar plots in Fig. 2c. The spectral distance between the longitudinal laser modes is $\Delta \lambda$ = 21 nm in this NW. With a NW length of L = 3.1 µm, a group refractive index of *n*_g_ ≈ 5.2 can be deduced using the relationship *n*_g_ = *λ*^2^_*L*_/2L∆*λ*_L_ with *λ*_*L*_ being the longitudinal laser emission wavelengths in vacuum for mode numbers *k* = 38 to 40 (according to *n*_g_L = *kλ*_*L*_/2). Mode distances ∆*λ* obtained from other lasing NWs on Au film plotted versus the inverse NW length (1/*L*) of the NWs are depicted in Supplementary Fig. S6 in the SI. As expected, the longitudinal mode distances are nearly proportional to 1/*L* with an average slope corresponding to a group index of 5.7. Deviation from this average value is due to the dependence of the group index *n*_g_(*d*) on the NW diameter. A comparison of the experimentally determined group indices with calculated *n*_g_ values using FDTD calculations (see Supplementary Fig. S13) indicate that only hybrid mode 2 (see Supplementary Fig. S7e) supports lasing in NWs on Au film. This is in agreement with FDTD threshold gain calculations, which reveal low plasmonic losses for the predominantly photonic mode 2, while the purely plasmonic mode 1 and other hybrid modes (e.g., mode 3) possess much higher plasmonic losses. The low loss hybrid mode 2 is an essential prerequisite to accomplish an E–field induced lasing modulation GaAs NWs where the Au film acts as a capacitor plate.

The energy positions of the emission peaks demonstrate that the actual lattice temperature in the excited NWs is significantly higher than the cryostat temperature of *T*_cryo_ = 77 K. A comparison with material gain calculations (see Supplementary Fig. S18) suggests a temperature of ~ 160 K inside the excited NWs considering both a temperature dependent^14^, and a carrier density dependent bandgap shrinkage^50,51^. A similar lattice temperature is observed in the photoluminescence spectra of NWs on glass and Au film and Au-coated NWs at moderate excitation power below lasing threshold (see Supplementary Fig. S15 for a NWs on Au and on glass). The higher lattice temperature is attributed to hot carrier relaxation and a high non-radiative surface relaxation rate in the GaAs NWs, which leads to a significant heat accumulation within the NWs during the emission process.

**Figure 3:**
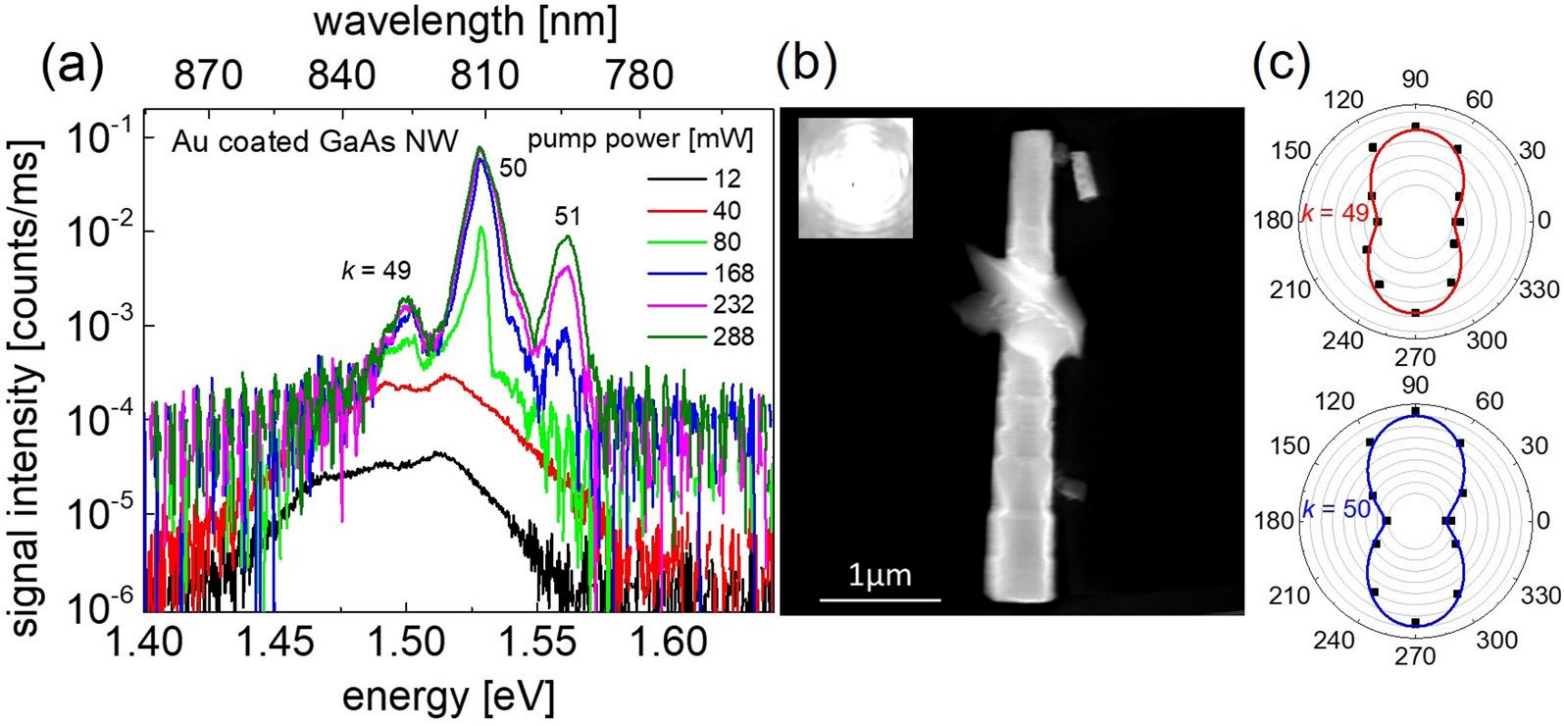
Lasing from an Au coated GaAs NW on glass substrate. (**a**) Pump power dependent lasing spectra of an Au coated GaAs NW on glass substrate at *T*_cryo_ = 77 K. (**b**) SEM image of the investigated NW. The inset shows the optical image of the lasing NW at 120 mW. (**c**) Polar plots of longitudinal lasing modes *k* = 49 and 50.

Au-coated GaAs NWs on glass substrate show similar lasing spectra as NWs on Au film. Figure 3a shows the pump power dependent spectra of an Au-coated NW with a length of 3.6 µm and tip and base diameters of 290 and 540 nm, respectively (NW #7 in Supplementary Table ST3 and ST6). The SEM image of the Au-coated NW is displayed in Fig. 3b. The inset in Fig. 3b shows the optical image of the NW emission at a pump power of 120 mW. Polar plots of two longitudinal lasing modes (*k* = 49 and 50) are demonstrated in Fig. 3c. The threshold power for lasing is higher than for NWs on Au film. This is partially caused by higher plasmonic losses in this coaxial configuration at the NW lasing energy but mainly originates from a reduced pump photon absorption in the GaAs NW core because of pump dissipation in the Au shell (see SI Sect. 3(g)). The spectral distance of *k* = 49 to 51 longitudinal modes in this Au-coated NW is ∆*λ* = 16.3 nm resulting in a group refractive index of *n*_g_ = 5.6. The longitudinal mode distances ∆*λ* of other Au-coated NWs versus the inverse NW length (1/L) are shown in Supplementary Fig. S6. The experimentally obtained group indices and calculated *n*_g_ values using FDTD calculations are presented in Supplementary Fig. S13. In contrast to the NWs on Au film, the waveguide modes HE11a/b and TE01 can support lasing. For tip diameters smaller than ~ 260 nm (including the Au coating), there is a preference for the HE11a/b modes because of their higher facet reflection compared to TE01 mode. For larger tip diameters, the facet reflection and confinement factor Γ of the TE01 mode exceed the values of the HE11a/b modes and thus becomes the supported lasing mode. Mode profile calculations (see Supplementary Fig. S7) of these effective medium (EM) air-Au/Al_2_O_3_/GaAs NWs indicate that the hybrid HE11a/b or TE01 modes have a weak plasmonic character with moderate dissipative losses. This property makes Au-coated NWs ideal for the design of NW lasers with electromagnetic or conductive shielding due to the plasmonic near field coupling of adjacent Au nanoparticles. Asymmetrically (half-sided) Au-coated NWs further offer prospects for electro-optic modulation or for plasmonically induced extrinsic chiral emission^44^. The latter could be utilized to control the polarization of NW lasing devices as has already been demonstrated in different plasmonic NW designs^52–54^.

**Figure 4:**
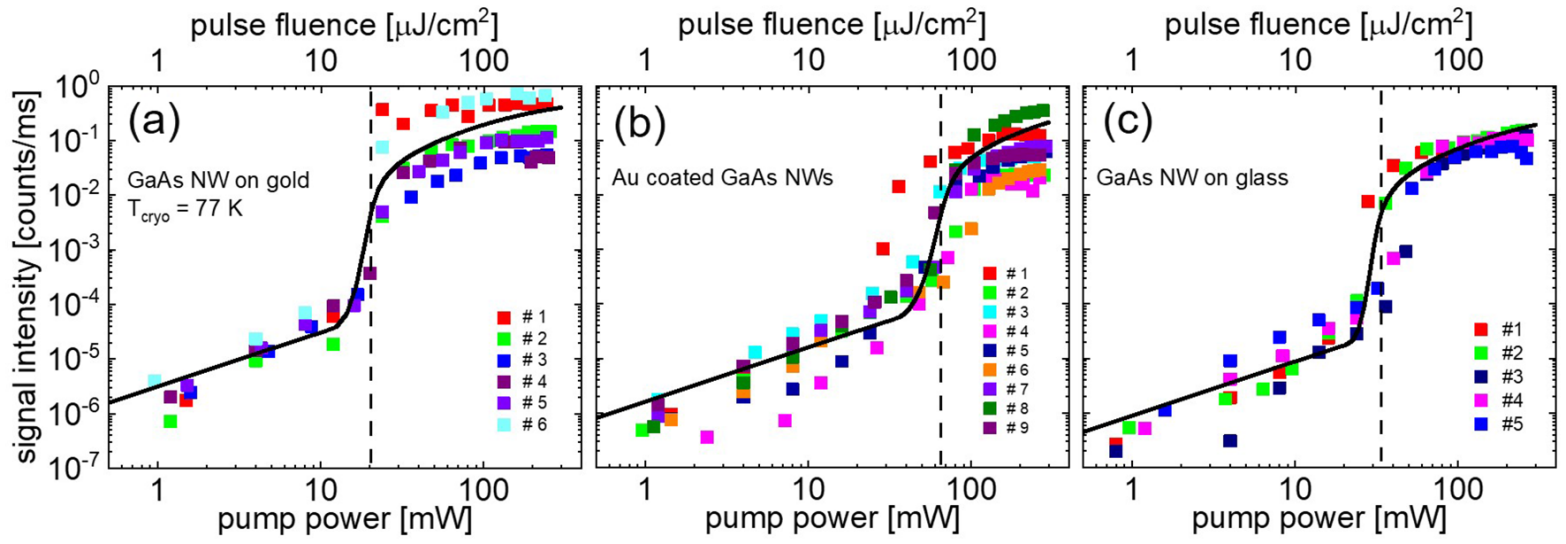
Peak power of the NW emission versus pump power (*L*–*L* plot) obtained from lasing spectra of (**a**) NWs on Au film, (**b**) Au-coated NWs and (**c**) NWs on glass at *T*_cryo_ = 77 K. Different colored symbols indicate NWs with different dimensions, which are summarized in tables ST1 to ST3. The thick black line shows calculated *L*–*L* curves of “average” NWs defined in table ST7. The vertical dashed lines indicate the approximate threshold power necessary for lasing.

To analyze the lasing behavior in the different NW configurations, we calculated the output laser power versus excitation pump power (*L–L* plot) by applying a coupled rate equation model for the photo-generated carrier density *N* and emitted cavity photons *S.* As one extension to previous models^13,55^, the onset of pump pulse transparency at high intensities, when the energy difference between the quasi-Fermi levels starts exceeding the exciting laser energy, was taken into account (see SI Sects. 7 and 8). Secondly, we considered that the heavy-hole to conduction band transition is essentially blocked at the excitation wavelength of 720 nm because the energy position of the Fermi-level lies within the valence band (see SI Sect. 3(g)). A detailed description of the model calculation is given in the SI Sect. 8. Figure 4 shows the *L–L* plots of the laser peak power (symbols) extracted from power dependent lasing measurements at *T*_cryo_ = 77 K for different NWs (a) NWs on Au film, (b) Au-coated NWs and (c) NWs on glass as reference. The dimensions of the NWs are specified in Supplementary Tables ST1 to ST3. The experimental data in Fig. 4 is not normalized and the peak signal intensity is given in counts per millisecond. In the SI Sect. 6 it is shown that a longitudinal mode laser line with a peak signal intensity of 0.1 counts/ms emits a power of approximately 1.7 µW. The full black lines in Fig. 4a–c present the calculated *L–L*-curves for an average “model” NW for each configuration. Calculated parameters of these average NWs are given in Supplementary Tables ST4 to ST6 (highlighted in the last row). The fitted threshold gain values obtained in the *L–L* model are 2500, 2900 and 1900 cm^−1^ for NWs on Au film, Au-coated NWs and NWs on glass, respectively. These values are in fair agreement to the FDTD values for the “average” model NWs (2200, 2000 and 700 cm^−1^). Deviations between these gain values and fitted values from the *L–L* plots are mainly attributed to imperfect end facets and scattering losses along the NW surface which were not considered in the FDTD simulations. Both effects are particularly significant for the TE01 mode which has the maximum of its *E*-field distribution in the vicinity of the NW surface (see Supplementary Fig. S7).

The *L–L* calculations reproduce the experimentally observed trend that NWs on Au film require the lowest threshold power for ASE and lasing (~ 20 mW, corresponding to a pulse fluence of ~ 20 μJ/cm^2^, indicated by dashed vertical lines in Fig. a) despite higher plasmonic losses than for NWs on glass, followed by NWs on glass (~ 35 mW, corresponding to ~ 35 μJ/cm^2^) and Au-coated NWs (~ 65 mW, corresponding to ~ 65 μJ/cm^2^). FDTD calculations reveal that the advantageous effect of a reduced threshold power for NWs on Au film is caused by an increase of the absorbed pump power *η*_p_ in this configuration because of the plasmonic E-field enhancement effect. The *L–L* analysis and FDTD calculations further reveal that the increased threshold power for Au-coated NWs is only weakly caused by the increased plasmonic losses in this coaxial structure but mainly originates from a reduced absorbed pump power *η*_p_ in the GaAs NW core because of pump absorption in the Au coating (see also SI, Sect. 3 (g)). At higher pump power, the NW laser intensity starts to saturate in all configurations, which is attributed to the increasing influence of Auger recombination^13,14^ and to the onset of pump pulse transparency.

### Characterization of lasing up to room temperature

**Figure 5:**
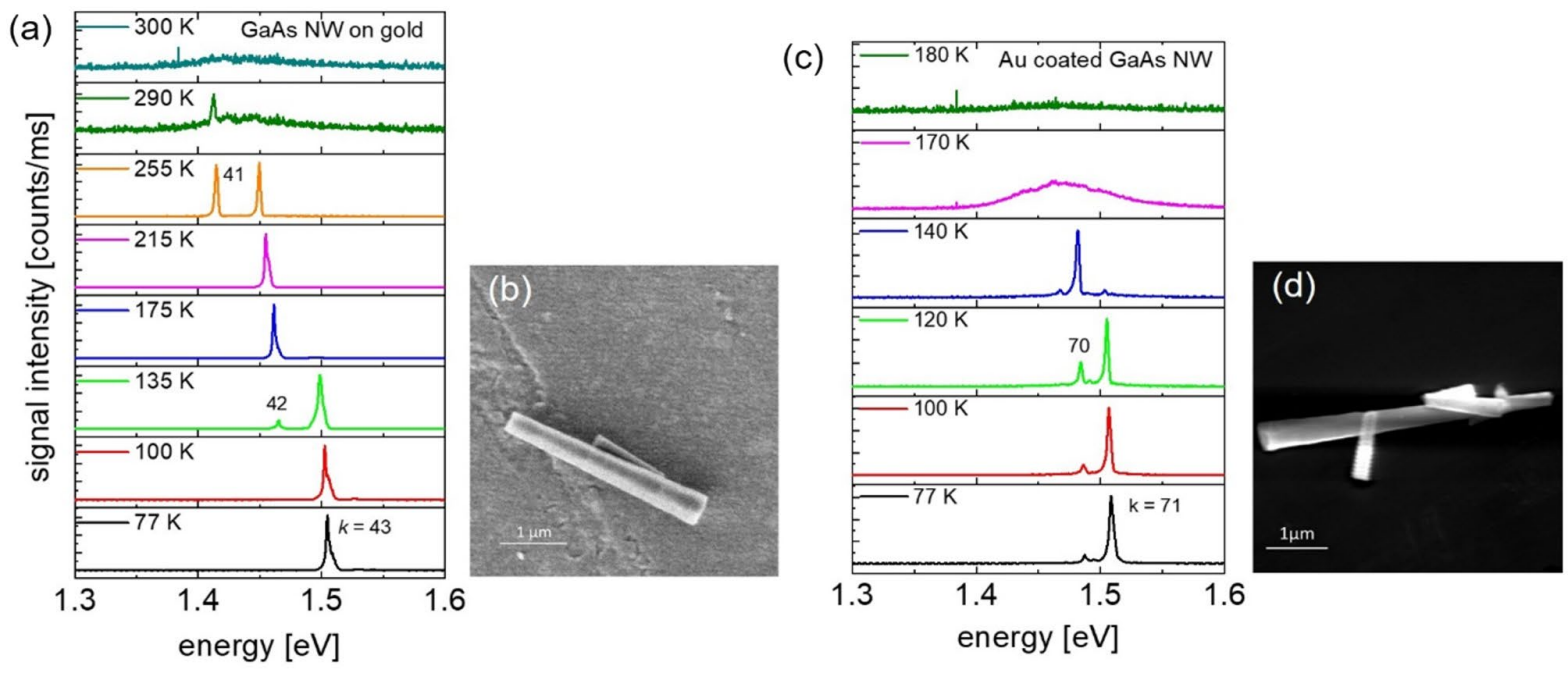
Temperature dependent laser emission spectra of (**a**) a GaAs NW on Au film at an excitation power of 120 mW and (**c**) an Au coated NW on glass substrate at a pump power of 140 mW. The redshift and weakening of the laser emission with increasing temperature is attributed to temperature-induced changes of the refractive index and the gap energy as well as a reduced material gain. The SEM images of the investigated NWs are shown in (**b**,**d**).

Based on the low temperature results we investigated NWs on Au film and Au-coated NWs at temperatures ranging from *T*_cryo_ = 77 K up to room temperature (295 K). Figures 5a,c show temperature dependent lasing spectra from a NW on Au film (*L* = 3.2 µm, *d*_t_ = 300 nm, *d*_b_ = 483 nm) and from an Au-coated NW (*L* = 4.9 µm, *d*_t_ = 238 nm, *d*_b_ = 491 nm) at a pump power of 120 and 140 mW, respectively. Figures 5b,d show the SEM images of the investigated NWs. A polarizer was used to confirm the two lasing peaks correspond to longitudinal modes according to group index values of *n*_g_ = 5.6 and 6.0, respectively. The longitudinal lasing modes show a weak red-shift when the temperature is increased above 77 K. The red-shift is explained by an increase of the refractive index in the GaAs NW with increasing temperature^56^. The change of the emission wavelength is caused by the temperature induced bandgap shrinkage, which shifts the gain spectrum to lower energy and allowing modes with lower *k* number to emerge in the emission spectrum while modes with higher *k* number disappear. For most NWs, the lasing intensity becomes very weak or even stop lasing due to increasing loss at higher temperatures. This overall reduction in laser intensity is caused by the reduced material gain at a higher temperature (see Supplementary Fig. S18). Increasing non-radiative losses due to carrier capture at surface traps or at lattice imperfections further contribute to the reduction of the laser intensity.

**Figure 6:**
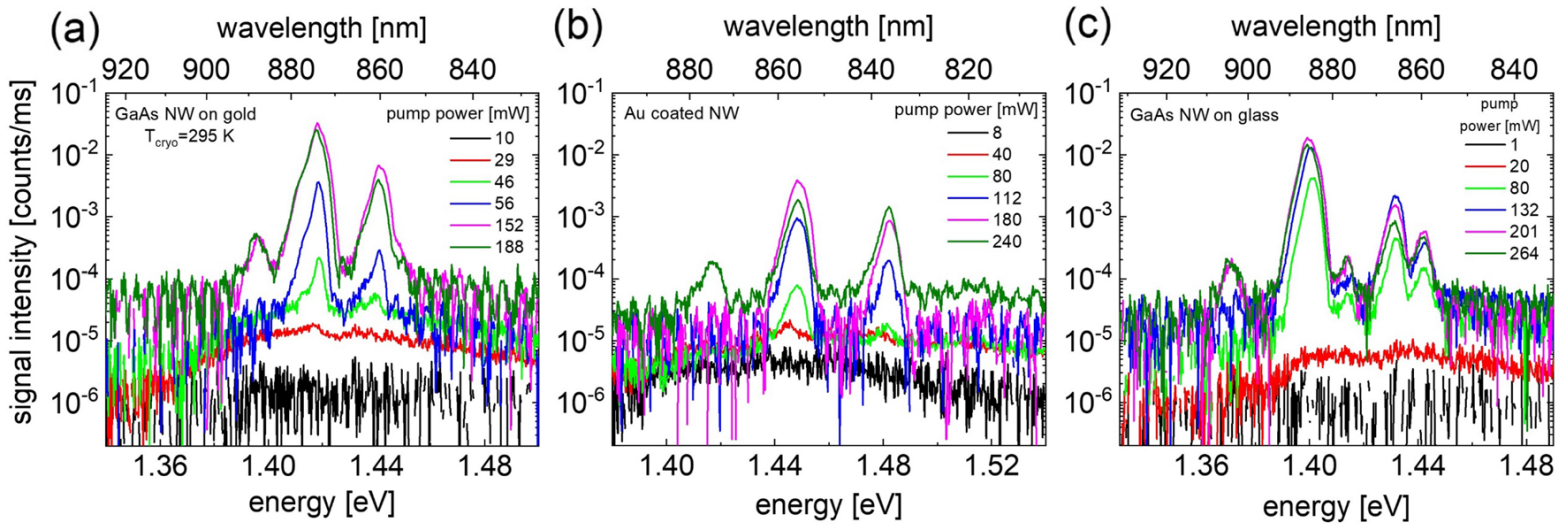
Pump power dependent lasing. (**a**) Spectra at *T*_cryo_ = 295 K of a NW on Au film (**b**) an Au coated GaAs NW on glass and, (**c**) GaAs NW on glass at *λ*_p_ = 720 nm.

For power dependent lasing experiments at room temperature, we transferred the NWs to the substrate with the high-density method 1 (see above and SI Sect. 1(b)), which resulted in several NWs that still showed lasing. Figures 6a,b show the power dependent lasing spectra from a single NW on Au substrate and from a single Au coated NW at *T*_cryo_ = 295 K. In case of GaAs NWs on glass substrate (Fig. 6c), more than two lasing NWs were present within the excitation laser spot. Polarization dependent measurements indicated that the longitudinal modes at 1.399 and 1.431 eV were from the same NW on glass. Since an ex-post identification of the lasing NWs with SEM was no longer possible, the NW dimensions were estimated from the longitudinal mode distances Δ*λ*_L_ in the lasing spectra. A comparison with Fig. S6 revealed a length of *L* $\approx$ 4.2 µm and a tip and bottom diameter of *d*_t_ $\approx$ 300 nm and *d*_b_ $\approx$ 500 nm for the NW on Au, *L* $\approx$ 3.0 µm and *d*_t_ $\approx$ 350 nm and *d*_b_ $\approx$ 500 nm for the Au coated NW and *L* $\approx$ 3.0 µm and *d*_t_ $\approx$ 350 and *d*_b_ $\approx$ 500 nm for the NW on glass substrate. FDTD calculations provided threshold gain values of 1790 and 1800 cm^−1^ for the NW on Au substrate and for the Au-coated NW, respectively, as well as 860 cm^−1^ for the NW on glass.

**Figure 7:**
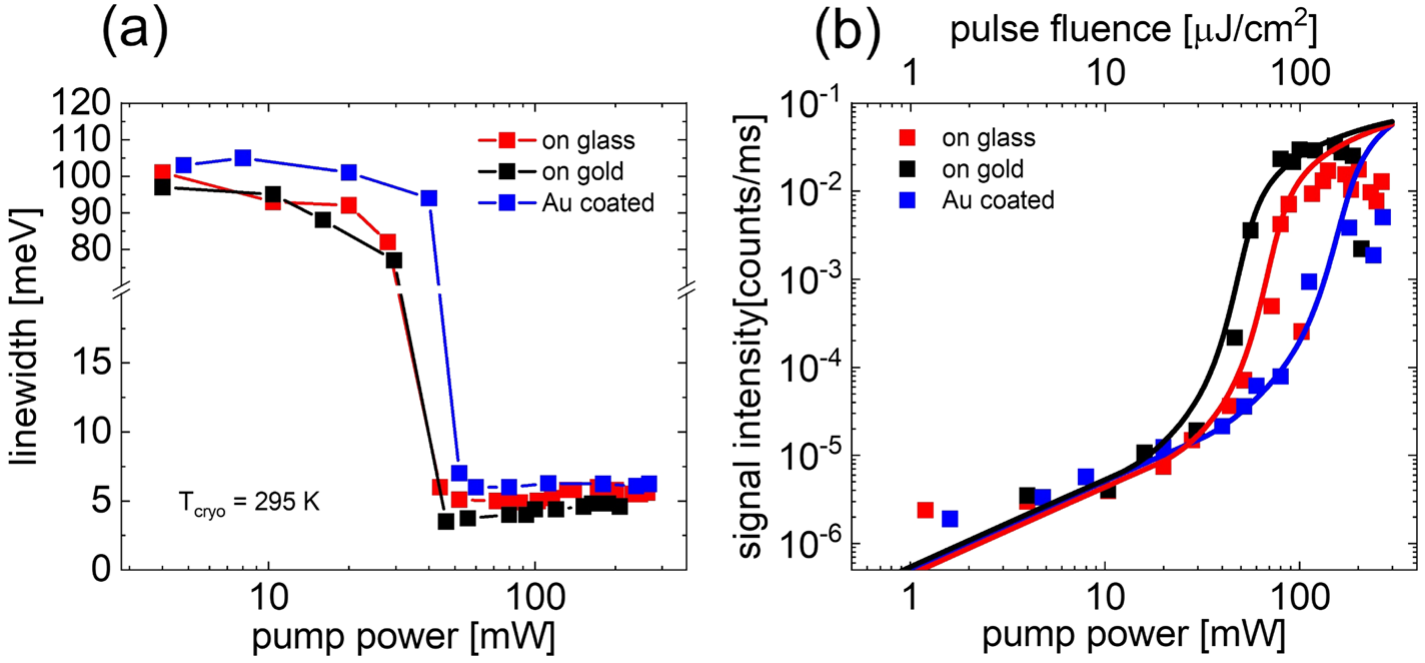
(**a**) Linewidth versus pump power extracted from the lasing spectra in Fig. 6 of a single GaAs NW on glass (red squares), on Au film (black squares) and from an Au coated NW (blue squares) at *T*_cryo_ = 295 K. (**b**) *L*–*L* plots obtained from lasing spectra in Fig. 6. The black, red, and blue lines show calculated *L*–*L* curves.

Figure 7a shows the emission linewidths versus pump power extracted from Figs. 6a–c. The linewidth of the spontaneous emission (photoluminescence) below threshold for all samples is approximately 100 meV. The linewidth decreases when the excitation power approaches the lasing threshold and drastically reduces to 4 to 6 meV beyond threshold indicating the onset of lasing. The linewidths of the longitudinal lasing modes are smallest for the NW on Au substrate ranging from ~ 4 to 5 meV. Linewidths for the NWs on glass are ranging from ~ 5 to 6 meV, and are approximately 6 meV for the Au coated NW. The smaller linewidth for the NW on Au substrate suggests that the escape rate of photons is somewhat smaller compared to that for the NW on glass and for the Au coated NW. This observation is consistent with the earlier statement that the lasing hybrid mode 2 is less sensitive to end facet and scattering losses along the NW surface than the TE01 mode in NWs on glass or for Au coated NWs.

A closer look at the spectra in Fig. 6 and linewidth plot in Fig. 7a further reveals that the laser lines slightly broaden with increasing pump power and show a small shoulder at the low energy side. Similar features have been observed earlier by other groups as well ^17,39^. A possible explanation for this effect is that the pulsed pump laser source creates a dynamically changing carrier density which shifts and broadens the gain profile during the transient excitation. Increasing excitation densities enhance this effect. The visibility of the effect is probably more distinct in the NW on Au sample because of the smaller mode linewidth compared to the other NW samples.

The *L–L* plots of the laser peak intensities (extracted from Fig. 6) are shown as black symbols for the NW on Au film and as blue symbols for the Au coated NW in Fig. 7b. The *L–L* plot for the NW on glass is shown as red symbols for reference. The drop of the signal intensity at highest pump powers in all three samples is attributed to a significant increase of the NW temperature and the onset of thermal deterioration. The full black, blue and red lines are calculated *L–L*-curves for the corresponding NW. For the calculations we applied the coupled rate equation model described in the SI Sect. 8. In these calculations, we used the same parameters as for the 77 K calculations with the exception of an enhanced absorption cross-section since the heavy-hole to conduction band transition is more efficient at room temperature. The fitted threshold gains obtained in the *L–L* model are 3100, 3400 and 2800 cm^−1^ for the NW on Au film, for the Au-coated NW and for the NW on glass, respectively. These *g*_th_ values significantly exceed the fitted values for NWs lasing at 77 K, which supports our earlier interpretation that increasing non-radiative losses due to carrier capture at traps or at lattice imperfections are responsible for the reduction of the laser intensity at room temperature. We expect that an AlGaAs surface passivation shell around the GaAs NWs will lead to a significantly reduced lasing threshold at room temperature.

## Summary

We have investigated optically pumped lasing in highly Zn-doped Al_2_O_3_/GaAs NWs on Au film and in NWs coated with Au nanoparticles. Both types of hybrid Au/GaAs NW structures exhibit lasing up to room temperature.

NWs on an Au film show a lower lasing threshold than NWs on glass despite surface plasmon dissipation. This reduction is due to an enhanced absorption cross-section caused by the presence of the Au film. FDTD simulations and *L-L* calculations indicate that lasing of NWs on Au film is supported by a low loss hybrid mode with nearly photonic character. Both advantages make highly Zn-doped GaAs NWs on noble metals suitable for electro-optic laser modulation where the Au film provides a plasmonic E-Field enhancement and simultaneously serves as a capacitor plate.

For Au-coated NWs, FDTD simulations confirm hybrid lasing with moderate plasmonic losses if the Au layer around the NW does not form a continuous film but isolated Au nanoparticles instead. The *L–L* analysis reveals that the increased threshold power for these Au-coated NWs is mainly caused by the dissipation of pump light within the Au-coating, which can be reduced by decreasing the Au-to-air filling factor. The coaxial design offers the prospect for electromagnetic wave shielding due to a plasmonic near field coupling of adjacent Au nanoparticles. Au-coated NWs are potentially appropriate for electrooptical applications and polarization-controlled NW lasing devices.

Our investigations in using GaAs NWs with Au film/particles operating in mainly photonic modes open new prospects for on-chip integration of nanolasers with new functionalities combining the advantages of both plasmonic effects and low loss photonic lasing.

## Methods

A similar experimental setup as described in^28^ was used: Ultrashort ~ 150 fs laser pulses provided by Ti–Sapphire laser with a repetition rate of 80 MHz were used as the excitation source. The center wavelength of the laser pulses was tuned to 720 nm to excite the GaAs NWs slightly above the bandgap energy. For excitation density dependent measurements, the laser power was varied with neutral density filters. A long working distance 5X microscope objective with a numerical aperture of 0.14 NA focused the laser beam onto the NWs on the substrate. The samples were placed in a temperature-controlled cryostat on XYZ translation stage for temperature dependent lasing experiments ranging from 77 K to room temperature (295 K). The spot size of the laser focus was approximately 40 µm in diameter. The emission from the NW samples was collected using the same microscope objective. A short-pass dielectric mirror with high reflectivity at the laser emission wavelength directed the NW emission into a compact CCD spectrometer. Additional band pass filters with a cut-off wavelength of 750 nm were placed in front of the spectrometer to suppress the excitation laser background at 720 nm. A sheet polarizer in front of the spectrometer analyzes the polarization direction of the NW emission. The beam of the NW lasing emission was directed into a CCD camera with a flip-mirror to provide an image of the lasing NWs within the optically pumped area. The resolution of the image setup was not sufficient to determine the length of the lasing NWs but was sufficient to visualize and count the number of lasing and non-lasing NWs within the focus diameter.

## Supplementary Information

Supplementary Information.

## Data Availability

The datasets supporting the conclusions of this article are included within the article and in the Supplementary Materials.
