# Author Correction: Effect of Au substrate and coating on the lasing characteristics of GaAs nanowires

**DOI:** 10.1038/s41598-021-02531-5

**Published:** 2021-11-23

**Authors:** Gyanan Aman, Fatemesadat Mohammadi, Martin Fränzl, Mykhaylo Lysevych, Hark Hoe Tan, Chennupati Jagadish, Heidrun Schmitzer, Marc Cahay, Hans Peter Wagner

**Affiliations:** 1 Department of Electrical Engineering and Computer Science, University of Cincinnati, Cincinnati, OH 45221 USA; 2 Department of Physics, University of Cincinnati, Cincinnati, OH 45221 USA; 3 Department of Physics, University of Leipzig, 04109 Leipzig, Germany; 4 Department of Electronic Materials Engineering, ARC Center of Excellence for Transformative Meta-Optical Systems, Research School of Physics, The Australian National University, Canberra, ACT 2601 Australia; 5 Department of Physics, Xavier University, Cincinnati, OH 45207 USA

Correction to: *Scientific Reports* 10.1038/s41598-021-00855-w, published online 01 November 2021

The original version of this Article contained an error in the spelling of the author Fatemesadat Mohammadi which was incorrectly given as Fatemeh Mohammadi.

The original Article and accompanying Supplementary Information file have been corrected.

