# Supplementary material for: Effect of Au substrate and coating on the lasing characteristics of GaAs nanowires
Source: Sci Rep. 2021 Nov 1;11:21378. doi: 10.1038/s41598-021-00855-w (PMC8560920; doi:10.1038/s41598-021-00855-w)
Supplement: Supplementary file 1 — Supplementary Information. [file 41598_2021_855_MOESM1_ESM.pdf]

Supplementary Information

Effect of Au substrate and coating on the lasing characteristics of GaAs nanowires

Gyanan Aman[1], Fatemesadat Mohammadi[2], Martin Fränzl[3], Mykhaylo Lysevych[4], Hark Hoe Tan[4], Chennupati Jagadish[4], Heidrun Schmitzer[5], Marc Cahay[1], Hans Peter Wagner[1, 2]

[1] Department of Electrical Engineering and Computer Science, University of Cincinnati, Cincinnati, OH 45221, USA

[2] Department of Physics, University of Cincinnati, Cincinnati, OH45221, USA

[3] Department of Physics, University of Leipzig, 04109, Germany

[4] Department of Electronic Materials Engineering, ARC Center of Excellence for Transformative Meta-Optical Systems, Research School of Physics, The Australian National University, Canberra, ACT 2601, Australia

[5] Department of Physics, Xavier University, Cincinnati, OH 45207, USA

1. Design of GaAs nanowire lasers

(a) Growth process and morphology of GaAs nanowire lasers

The GaAs NWs were grown by metal organic vapor phase epitaxy (MOVPE) on semi-insulating GaAs (111) B substrates. During the growth process, the substrates were pre-treated with poly-L-lysine (PLL) and 100 nm Au colloidal solutions before placing them inside a horizontal flow reactor [1]. The PLL acts as a thin polyelectrolyte layer to electrostatically attract and immobilize the Au colloids on the substrate, which acts as a seed for GaAs nanowire growth. The growth process was executed at a high temperature of 575 ºC in presence of AsH_3 and trimethylgallium at a V/III ratio of ~1.4. The zinc doping with an acceptor concentration of $N_A = 2x10^{19}$ cm^{-3} changes the structure of NWs from a pure wurtzite crystal to a zincblende twining superlattice (TSL) crystal. More details

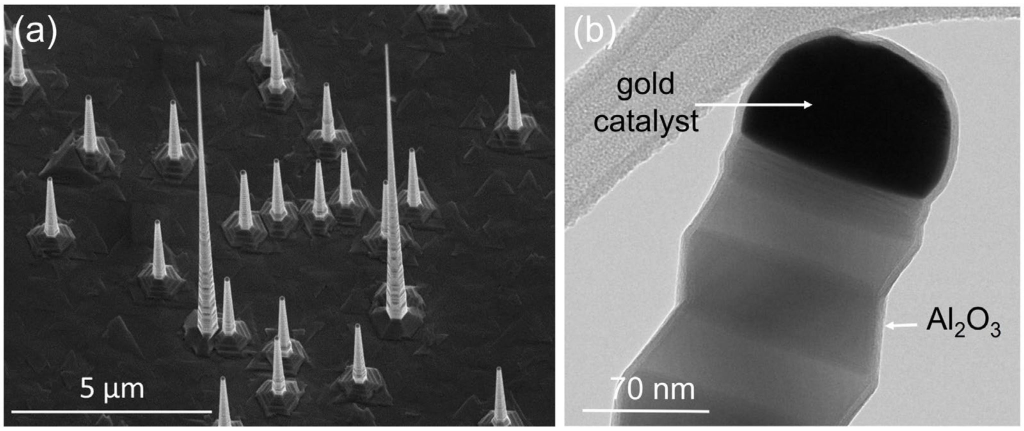

Figure S1: The structural characterization of GaAs NW. **(a)** SEM image of highly Zn doped GaAs NWs on GaAs (111) B substrate at the tilt angle of 30°. Most NWs have an average length of 3 µm and a mean diameter of 220 nm. A small fraction of NWs (5%) has a length of ~ 8-12 µm with a tip diameter of ~100-135 nm and a base diameter of ~510-570 nm. **(b)** TEM image of a single NW with Au catalyst on the tip and ~5 nm thick Al_2O_3 layer surrounding the NW. The alternating dark-bright contrast in both the SEM and TEM images throughout the entire NW indicates a zincblende twinning superlattice (TSL) structure.

on the growth process and optical properties of such NWs have been reported in Ref. [2]. The scanning electron microscope (SEM) image in Fig. S1 (a) shows the TSL structure of GaAs NW with alternating dark and bright contrast on the nanowire. The grown nanowires are hexagonal and conical in shape and most of the NWs have a mean diameter of 220 nm and length of 3 μm. A small fraction of ~5% of the NWs have a length of ~8-12 μm with a bottom diameter of ~510-570 nm and a tip diameter of ~100-135 nm. The investigation of lasing from broken off NWs with lengths ranging from ~2.5 to 6 μm and a core tip diameter ranging from 200 to 320 nm is the subject in this paper.

The GaAs NWs were subsequently coated with a 5 nm-thick Al_2O_3 layer by atomic layer deposition (ALD) to reduce the adsorption of atmospheric ions and metal induced band bending [3-5]. Figure S1 (b) shows the TEM image of a single nanowire with the Au catalyst and a 5 nm-thick Al_2O_3 layer grown around the nanowires. Some of these nanowires were nominally coated with ~10 nm thick Au layers by e-beam evaporation. Figure 1 (b) in the paper shows the TEM image of an Au-coated NW revealing that Au nanoplatelets and nanoparticles are formed rather than a continuous film. The Au nanoparticles range from 1 to 25 nm in size.

(b) Sample preparation

Three different designs of samples were investigated (i) Al_2O_3/GaAs NW on a glass substrate for reference (see sketch in Fig. S2), (ii) 10 nm Au/Al_2O_3/GaAs NW on a glass substrate, and (iii) Al_2O_3/GaAs NW on 200 nm-thick Au film on a glass substrate. The Au film was deposited on the glass substrate using an Organic Molecular Beam Deposition (OMBD) system. The growth temperature and growth rate of the Au film were 1150 °C and 0.014 Ås^{-1}, respectively.

The GaAs NWs are very fragile and break into pieces with different lengths and varying tip and

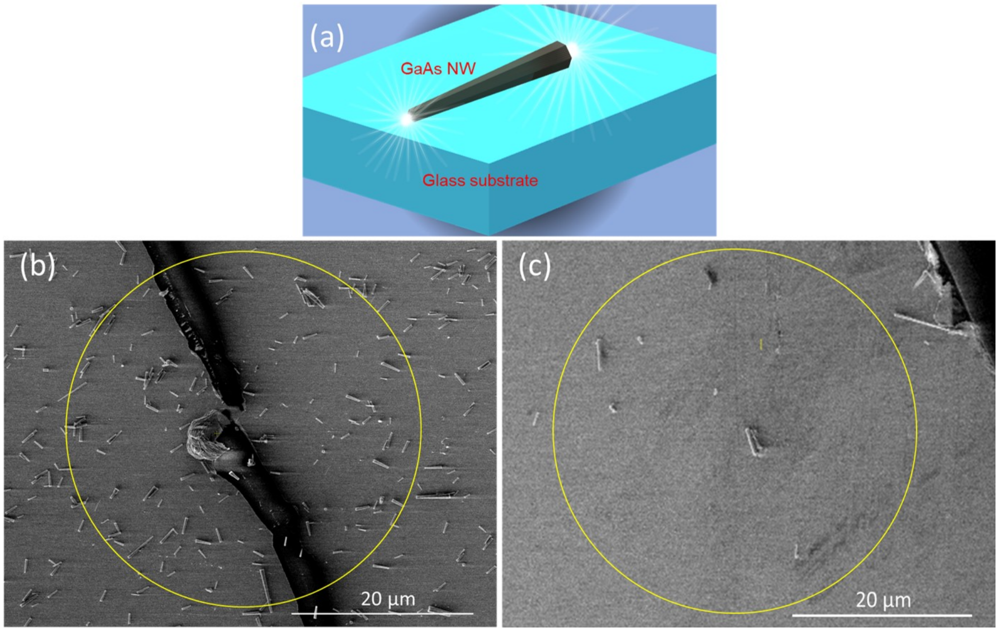

Figure S2: The sample preparation methods (1) and (2). **(a)** Schematic of a GaAs NW on the glass substrate. **(b)** SEM of GaAs NWs on the Au film showing the distribution of NWs prepared using method 1. **(c)** SEM of GaAs NWs on an Au film, showing the distribution of NWs prepared using method 2. The excitation spot size of the pump beam is indicated by the yellow circle.

base diameters when they are removed from the substrate. Two different methods were applied to transfer bare Al_2O_3/GaAs and coated Au/Al_2O_3/GaAs NWs to an Au film substrate and glass substrate: (1) Sliding the vertically oriented GaAs NWs over the glass and Au-coated substrate (method 1). (2) Using a thin brush to pick off and transfer GaAs NWs to the desired substrates (method 2). The two methods result in a significantly different nanowire density on the substrates. Figure S2 (b) shows the SEM image of GaAs NWs transferred by method 1, and Fig. S2 (c) is the SEM image of NWs transferred on an Au-coated glass substrate when method 2 was used for preparing the samples. Method 1 reveals a high NW density of more than 15 NWs within the excitation laser diameter of 40 μm, whereas method 2 provides a NW dispersion of 1 to 5 NWs within the excitation spot of the pump beam is shown as a yellow circle in Figs. S2 (b) and (c).

(c) Size distribution and identification of lasing NWs on the substrate

Due to the high density of nanowires on the samples prepared by method 1, it was impossible to reliably identify the lasing NW during lasing experiment with an optical microscope or an SEM

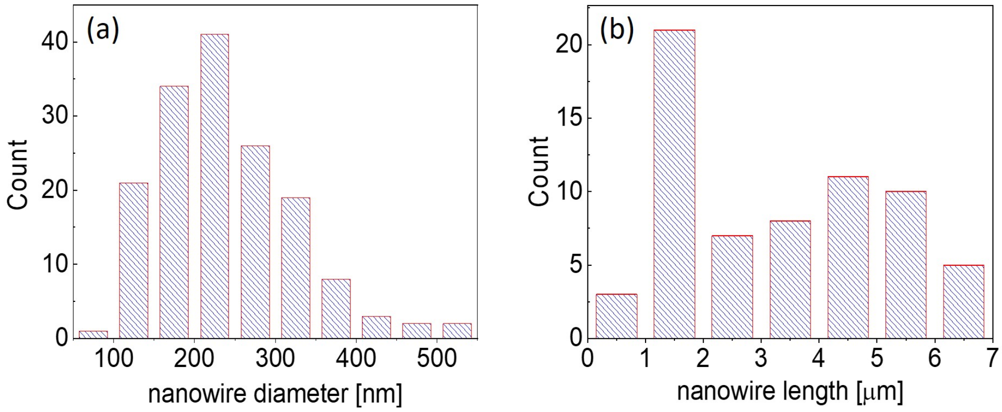

Figure S3: Distribution of the NWs. **(a)** mean diameters and **(b)** length on the substrate obtained by method 1. The distribution is obtained from the SEM of the NWs on the substrate.

afterwards. The much lower density provided by method 2 allowed us to unambiguously identify the lasing NW. SEM and optical microscopy investigations of samples that were prepared with method 1 and method 2 revealed 20% long (>3 μm) and thicker NWs (with a mean diameter >300 nm). Figure S3 (a) and (b) shows histograms of NW mean diameter and length, respectively, prepared with method (1). The length and tip diameter of the nanowires on three samples prepared with method 2 ranged between ~2.5 to 6 μm and ~200 to 350 nm. Most of the NWs lost their tip (including the Au catalyst) during the transfer. The short (smaller than 1 μm) broken off NW tips have mean diameters less than 150 nm which is below the optical diffraction limit and thus did not show laser emission in the experiments. In order to find the lasing NWs conveniently after the optical experiments using SEM imaging, they were deliberately dispersed near the edges of the substrate. During the lasing measurements, magnified camera images of the lasing NW at low and high power were captured to pinpoint the lasing NWs on the substrate. In the SEM, these NWs were located by correlating the images of the spot from the CCD camera to the calibrated magnification scale of the CCD image with the scale of the SEM image. Figure S4 (a) and (b) shows the CCD image of the lasing GaAs NWs on a glass substrate at 10 and 75 mW pump power, respectively. Figure S4 (c) shows the SEM image of the same spot.

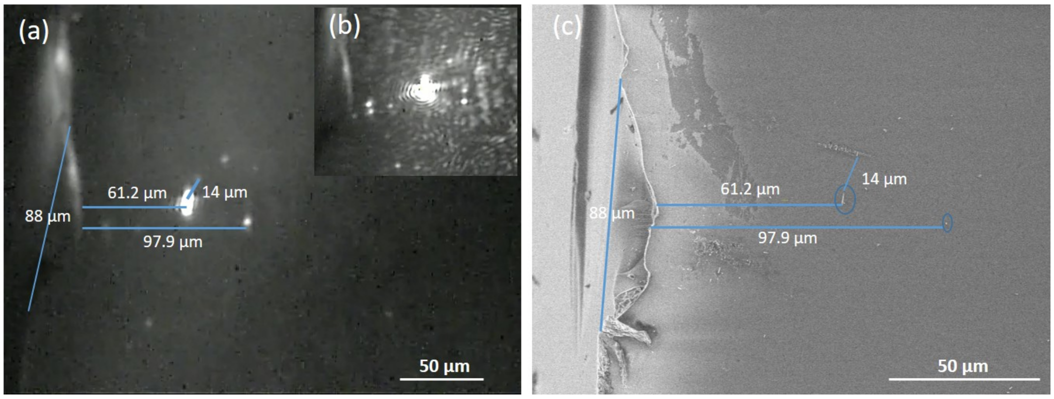

Figure S4: Identification of lasing NWs on the substrate. **(a)** CCD image of GaAs NW on glass at a cryostat temperature of 77 K and at a pump power of 10 mW. **(b)** Image of the lasing NW with interference pattern at a pump power of 75 mW. **(c)** SEM image of the same GaAs NW on glass captured at a magnification of 650x.

2. Nanowire laser characterization

The optical set-up used in the lasing experiments on the three types of samples has been briefly described in the paper. Figure S5 (a) shows a power dependent emission spectrum from a single GaAs NW on glass at a cryostat temperature of T_{cryo} = 77 K on a logarithmic scale pumped at λ_p = 720 nm. At a low pump power (less than 16 mW), the spectrum reveals a broad spontaneous emission band. At a pump power of 24 mW, several spectrally wide peaks emerge in the photoluminescence spectrum, which is attributed to longitudinal resonator modes of the amplified

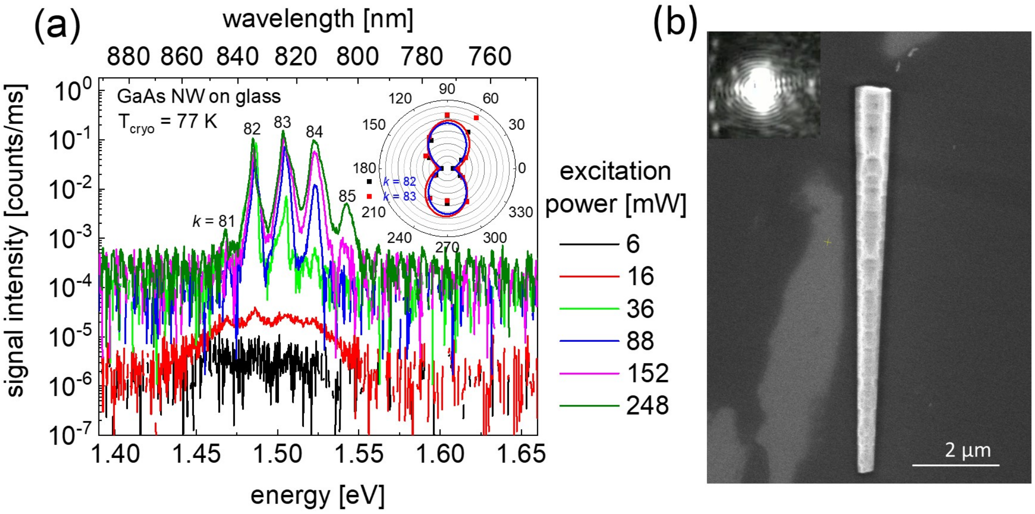

Figure S5 Lasing NW on a glass substrate **(a)** Power dependent lasing spectra of GaAs NW on glass measured at the cryostat temperature of 77 K at an excitation wavelength of 720 nm. The inset shows a polar plot of the lasing emission for different longitudinal modes. The solid lines in the inset are a guide for the eye. **(b)** SEM of the same lasing nanowire with a length of 6.1 µm, tip diameter of 250 nm and base diameter of 560 nm. The inset shows the interference pattern of the laser emission obtained from the NW on glass.

stimulated emission (ASE). These peaks get more distinct and spectrally narrower with increasing pump power and become the lasing modes. At a pump power of ~150 mW, the linewidth of the longitudinal laser modes is narrow and close to 1 nm (note the logarithmic scale). The energy position of the laser lines suggests a higher lattice and carrier temperature in the nanowires compared to the cryostat temperature due to a high non-radiative recombination rate at the NW surface. A comparison between the calculated and the experimentally observed photoluminescence spectra at 77 K cryostat temperature (see section S4) resulted in an actual NW temperature of T_{NW} = 160 K. The polarization of the emission peaks was analyzed by placing a polarizer in front of the entrance slit of the spectrometer. The polarization of longitudinal modes at 1.46, 1.48, 1.50 and 1.52 eV at 80 mW pump power is oriented along the NW long axis as shown in the polar plots in the inset of Fig. S5 (a).

Figure S5 (b) reveals the SEM image of the same lasing NW on the glass substrate. The SEM measurements of the NW gave a length L = 6.1 μm with a tip diameter of 250 nm and a base diameter of 560 nm (NW #2 in table ST1). The inset in Fig. S5 (b) depicts the camera image of the emission from the same nanowire at a pump power of 80 mW. It reveals an intense and distinct interference pattern, confirming coherent laser emission from the nanowire. The spectral distance between the longitudinal laser modes is $\Delta\lambda$ =10 nm in this NW. A group refractive index $n_g \approx 5.6$ was deduced using the relationship

$$n_g = \frac{\lambda_L^2}{2L\Delta\lambda} \tag{S1}$$

with λ_L being the longitudinal laser emission wavelengths in vacuum for mode numbers k = 81 to 85 (according to $n_g L = k\lambda_L / 2$). Mode distances $\Delta\lambda$ obtained from other lasing NWs on glass plotted versus the inverse NW length (1/L) of the NWs are depicted in Fig. S6.

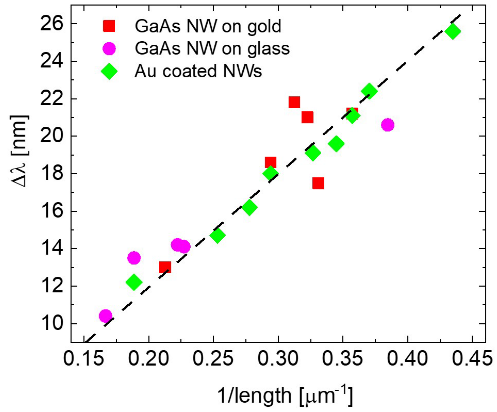

Figure S6: Experimentally observed spectral distance Δλ between longitudinal modes as a function of inverse NW length of all investigated GaAs NWs on glass (red square), GaAs NWs on Au film (pink circle), and Au-coated NWs on glass (green diamond).

The dimensions of all investigated NWs on the three types of samples characterized by SEM are summarized in tables ST1, ST2, and ST3. The experimentally determined tip and base diameters of the nanowires include the 5 nm-thick Al_2O_3 coating for bare NWs on glass and on Au film substrate. This adds a 10 nm/cos 30° = 11.55 nm coating length to the outer diameter (equal to two times the side length of the hexagon) of the core GaAs nanowire. In the Au-coated nanowires with

$\Delta\lambda$ obtained from all NWs versus the inverse NW length ($1/L$) of the NWs are shown in Fig. S6. As expected, the longitudinal mode distances are nearly proportional to $1/L$ with an average slope corresponding to a group index of 5.7. The deviations from this average value are due to the varying hybrid-plasmonic contributions of the lasing mode and because the dependence of group index $n_g(d)$ on the NW diameter (see section 3 (e)).

Table ST1: Dimensions of investigated nanowires on glass.

NW on glass	NW length [µm]	NW tip diameter [nm]	NW base diameter [nm]	Group index n_g
#1	4.4	296	520	5.5
#2	6.1	250	560	5.6
#3	2.6	240	340	6.6
#4	4.5	326	510	5.2
/#5	5.3	270	560	4.8

Table ST2: Dimensions of investigated nanowires on Au film.

NW on Au film	NW length [µm]	NW tip diameter [nm]	NW base diameter [nm]	Group index n_g
#1	3.1	300	485	5.2
#2	4.7	240	467	5.5
#3	3.02	254	380	5.9
#4	2.8	262	399	5.6
#5	3.5	317	501	5.2
#6	3.2	305	490	4.8

Table ST3: Dimensions of investigated Au-coated nanowires.

Au-coated NWs	NW length [µm]	NW tip diameter [nm]	NW base diameter [nm]	Group index n_g
#1	2.3	349	491	5.8
#2	5.3	230	506	5.2
#3	3.06	300	500	5.8
#4	2.8	328	490	5.8
#5	2.7	351	492	5.5
#6	3.95	278	470	5.7
#7	3.6	290	540	5.6
# 8	3.4	351	560	5.4
#9	2.9	310	455	5.9

3. Modeling of the NW waveguide parameters using FDTD calculations

Various parameters of the lasing nanowires such as waveguide mode profiles, effective refractive indices n_{eff}, group refractive indices n_g, plasmonic losses α_p, absorption cross sections σ_{abs}, reflectivity losses α_R and confinement factors Γ of bare NWs on glass and on Au and of Au-coated NWs were calculated with finite-difference time-domain (FDTD) simulations (Lumerical *Mode Solution,* and *FDTD Solution* packages), using material parameters of glass [6], Al_2O_3 [6], GaAs [6] and Au [7] at room temperature. In these simulations, un-tapered hexagonal GaAs NW were used. The outer diameters of the NWs ranged from 100 to 600 nm and the lengths L ranged from 1 to 6 µm.

The waveguide modes in uncoated and in Au-coated hexagonal GaAs NWs are similar to the guided modes in an air-clad dielectric cylinder; hence a similar mode convention is used for describing various modes. In our FDTD calculations, we consider 6 modes (HE11a, HE11b, TE01, HE21a, HE21b, TM01, see also E-intensity distributions in Fig. S7). The letters "HE" in the mode type refers to hybrid modes, "TE" represents transverse electric, and "TM" denotes transverse magnetic. The subscripts 'a' and 'b' indicate the two-polarization states of the same mode, parallel or normal to the substrate surface. The numbers "11" or "12" describe the radial order and angular symmetry of the modes [1]. In GaAs NWs on Au film, 4 of the 6 waveguide modes are plasmonic or

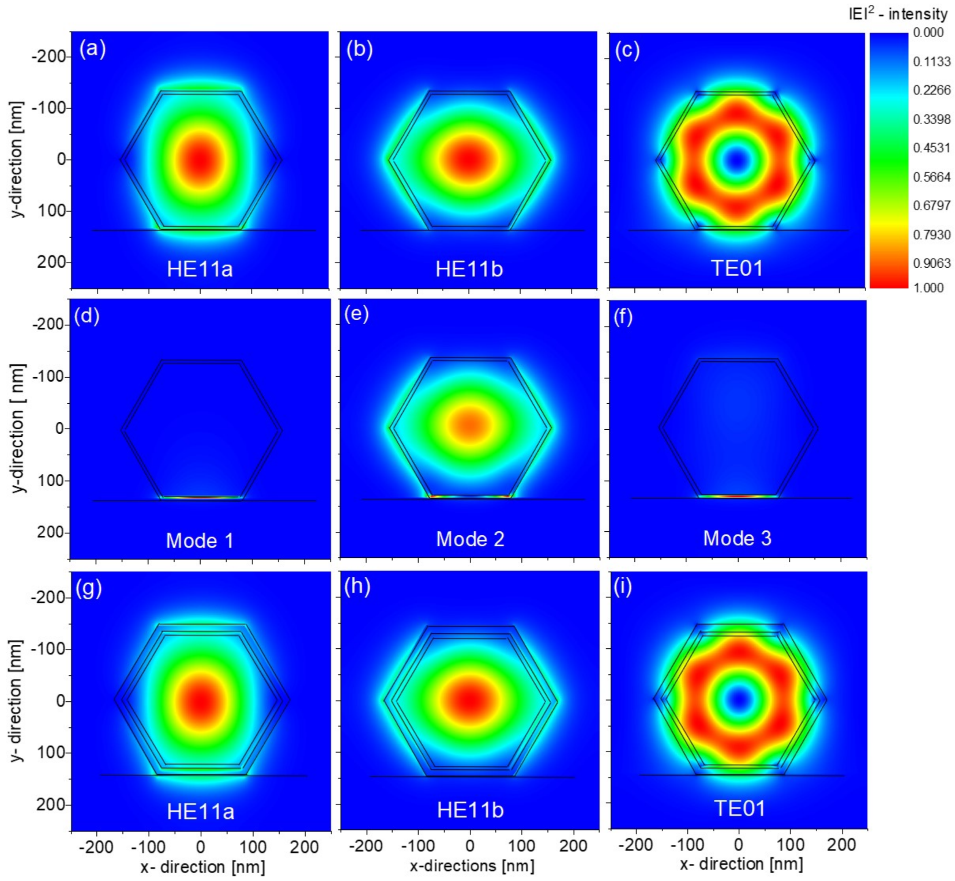

Figure S7: |E|^2 intensity distribution in a NW. **(a)**, **(b)**, and **(c)** Calculated |E|^2 intensity of 3 waveguide modes (HE11a, HE11b, and TE01) for GaAs NW coated with 5 nm thick Al_2O_3 on glass substrate. **(d)**, **(e)**, and **(f)** Mode profiles of Al_2O_3/GaAs NW on a 200 nm Au film. **(g)**, **(h)**, and **(i)** Mode profiles of Al_2O_3/GaAs NW with a 10 nm air/Au (p = 0.33) effective medium.

hybrid plasmonic modes. Two modes are mainly photonic modes. We enumerate these modes according to the descending magnitude of their effective refractive index values (mode 1 to 6).

(a) Waveguide mode calculations

The FDTD calculations (see below) revealed that for a GaAs core tip diameter of less than ~320 nm (which was the case in our investigated NWs) only the first three waveguide modes needed to be considered for lasing. The electric field profiles of the guided modes in a hexagonal GaAs NW with a diameter of 300 nm in the three configurations is demonstrated in Fig. S7. In all designs, the optical gain is provided by the GaAs NW core, while the Au nanoparticle shell around the NW and the Au film for the NW on Au film substrate contribute to optical losses because of plasmonic dissipation.

Figures S7 (a), (b), (c) show the supported photonic modes HE11a, HE11b, TE01 in GaAs NW coated with 5 nm thick Al_2O_3 on the glass substrate. It will be shown later that, depending on the tip diameter of the investigated NWs, the lasing mode is either a HE11a/b or a TE01 mode.

Guided mode simulations of Al_2O_3/GaAs NWs lying on a 200 nm thick Au-coated glass substrate are shown in Figs. S7 (d), (e), and (f). Mode 1 and mode 3 show a plasmonic character with the E-field being predominantly confined in the Al_2O_3 layer. Waveguide mode 2 shows a predominantly photonic behavior with a small plasmonic contribution at the NW/Au interface. In section 3(e) it will be shown that mode 2 is the preferred lasing mode for all investigated NWs on Au.

The profiles in Figs. S7 (g), (h), and (i) show the guided modes in the GaAs NW coated with a 5 nm-thick Al_2O_3 layer and a 10 nm Au-air (p = 0.33) effective medium (EM) around the NW. The modes HE11a, HE11b and TE01 in these coaxial structures are predominantly photonic.

(b) Effective refractive index (n_{eff}, κ)

Figure S8 shows the real part of the refractive index $n(d)$ as a function of the outer diameter d (which is two times the length of a hexagon side) for various guided modes supported in an untapered Al_2O_3/GaAs NW on glass. (The NW diameter includes the Al_2O_3 layer.) The NW diameter ranges from 100 to 600 nm. The waveguide mode calculations are performed at a wavelength of λ = 880 nm (~1.41 eV), which is a typical NW lasing wavelength at room temperature. The material parameters of GaAs, Al_2O_3, and SiO_2 in the simulations are from Palik et al. [8]. Different modes (HE11a, HE11b and TE01 are shown in Fig. S7) provide light confinement at different NW cut-off diameters. Modes with refractive indices below the index value of glass (n ~1.5) leak into the substrate. The effective refractive index increases with nanowire diameter. Lasing GaAs NWs on glass provide optical gain instead of absorption at λ = 880 nm. Therefore, the imaginary part of the refractive index was set to κ = 0 for bare NWs on glass, assuming no further losses due to scattering at the NW surface or at the NW end facets.

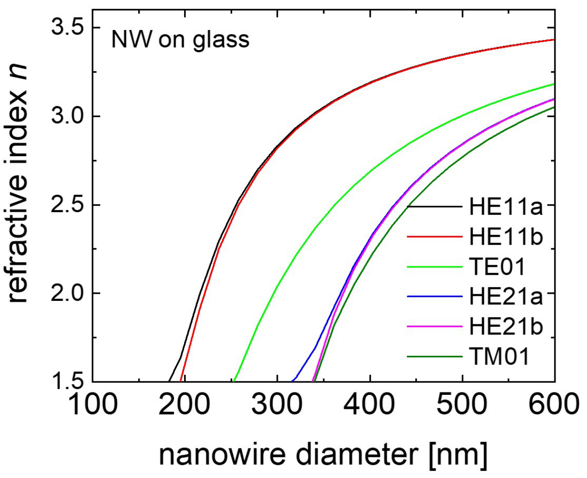

Figure S8: Effective index mode index as a function of the outer diameter of GaAs NW on glass substrate.

Figures S9 (a) and (b) show the real and the imaginary part of the refractive index $n(d)$ and $\kappa(d)$ as a function of the outer diameter for an Al_2O_3/GaAs NW on Au film. The complex refractive index of the Au film at room temperature is taken from Johnson and Christy [7]. The mode cut-off diameter is reached when the refractive index falls below the value of air ($n_{air} = 1$). When a GaAs NW is placed on the Au layer the imaginary part of the effective refractive index $\kappa(d)$ needs to be considered. The κ values shown in Fig. S9 (b) indicate the plasmonic or Ohmic losses occurring at the metal surface. Large plasmonic losses imply high threshold gain values g_{th} for lasing. The loss of plasmonic mode 1 (see Fig. S7) is high over the entire NW diameter range preventing plasmonic

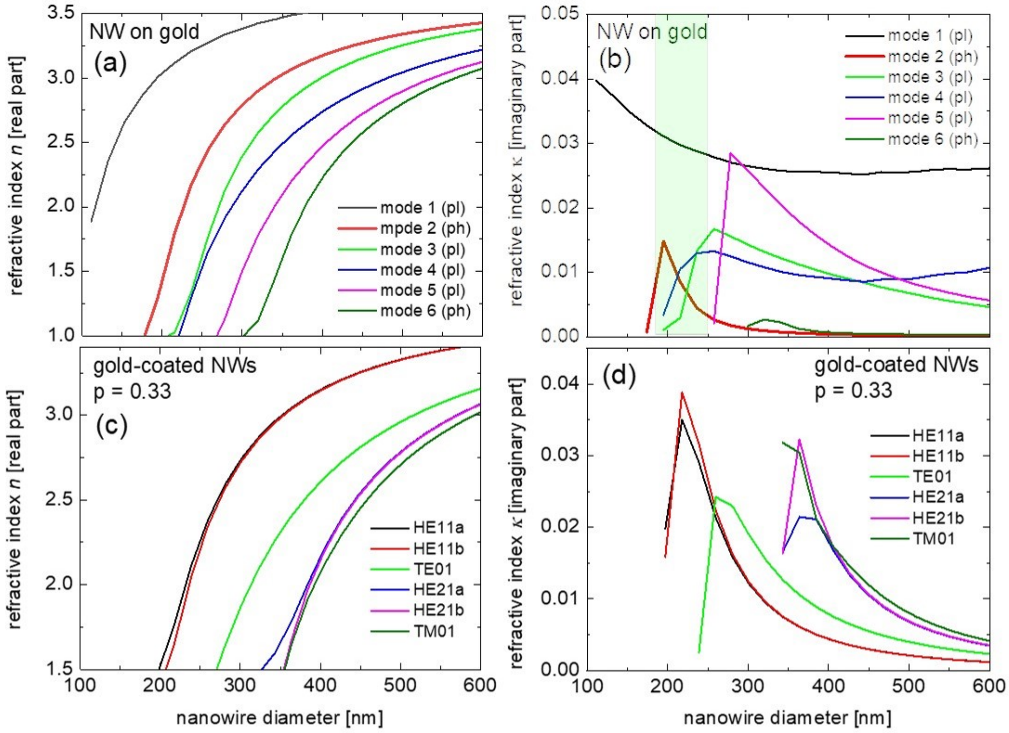

Figure S9: The effective refractive index of NWs. **(a)** and **(b)** real and imaginary parts of the refractive index as a function of the NW diameter for GaAs NW on Au. **(c)** and **(d)** real and imaginary part of the refractive index as a function of the diameter for Au-coated GaAs NW on glass substrate.

lasing. Other modes show lower plasmonic loss. Above the cutoff diameter of ~180 nm but below ~250 nm, mode 2 is a hybrid plasmonic-photonic mode with moderate plasmonic losses as indicated by the shaded area in Fig. S9 (b). Above 250 nm diameter mode 2 becomes increasingly photonic (see also Fig. S7) showing low plasmonic losses. Modes 3 to 5 remain hybrid plasmonic-photonic modes with moderate losses throughout the entire diameter range. Mode 6 has again a predominantly photonic character. Modes 2 and 6 reveal the lowest κ values for nanowire diameters above 300 nm emphasizing that these modes oscillate when the NWs are lasing.

In the case of Au-coated NWs, we performed simulations using a 10 nm-thick granular air/Au effective medium (EM) layer (see Fig. 1 in the manuscript). Since the Au clusters are in close vicinity with each other, the localized surface plasmons of individual nanoclusters can interact with each other and can be approximately treated as surface plasmon polaritons (SPPs) in an air/Au effective medium layer [9]. The complex refractive index of the composite metallic Au/air granular film is determined using effective medium (EM) theory. The effective dielectric permittivity (ε_e)

of two dimensional composites is given by [10],

$$\varepsilon_e = \frac{1}{2}\left\{(2p-1)(\varepsilon_m - \varepsilon_d) \pm \sqrt{(2p-1)^2(\varepsilon_m - \varepsilon_d)^2 + 4\varepsilon_m \varepsilon_d}\right\} \qquad \text{(S2)}$$

where ε_m is the permittivity of Au, ε_d is the permittivity of the dielectric GaAs and p is the filling factor of the Au in the Au/air film. The sign in eq. (S2) needs to be chosen appropriately to result in a positive imaginary part of ε_e. Using a p ~0.33 Au filling factor (estimated from the TEM image shown in Fig. 2 in the main text) we obtain a complex refractive index $n + i\kappa = 2.22 + 0.244$ at a lasing wavelength of $\lambda = 880$ nm. This value has been implemented into the Lumerical program for calculating the effective refractive index, group index, facet reflection and confinement factor for Au-coated nanowires. Figures S9 (c) and (d) show the real and imaginary part of the refractive index $n(d)$ as a function of NW diameter d for a nominally 10 nm thick EM Au-coated Al_2O_3/GaAs nanowire with an Au-to-air filling fraction of $p = 0.33$ on glass. Figure S9 (c) reveals very similar refractive indices as for GaAs NW on glass (see Fig. S8). The cut-off diameters of the GaAs core, when the refractive index of glass is reached, are somewhat smaller than for uncoated NW on glass. The imaginary part κ of the refractive index for Au coated nanowires is shown in Figure S9 (d). Au coated GaAs NWs show a higher plasmonic loss than mode 2 in GaAs NWs on Au films, suggesting higher g_{th} values to facilitate lasing.

Due to the conical shape of the highly Zn doped GaAs nanowires the refractive index of a waveguide mode continuously changes within the nanowires as function of the nanowire diameter d. We therefore approximated the effective refractive index n_{eff} + iκ for a specific mode in a conically shaped NW by calculating the average value of functions $n(d)$ and $\kappa(d)$ i.e.

$n_{eff} = \int_{d_t}^{d_b} n(d)\delta d / (d_b - d_t)$ where d_t is the tip diameter and d_b is the bottom diameter of the truncated nanowire under investigation.

(c) Mode confinement calculations

The confinement factor Γ of a particular waveguide mode is given by

$$\Gamma = \frac{\frac{c\varepsilon_o n_a}{2}\int_{A_a} |E|^2 dxdy}{P_z}, \qquad \text{(S3)}$$

where c is the speed of light in vacuum, n_a is the effective refractive index of the guided mode, ε_0 is the vacuum permittivity and P_z is the power flow in the propagation direction. A_a is the active area in which gain is provided. In the confinement factor calculation for GaAs NWs on glass and

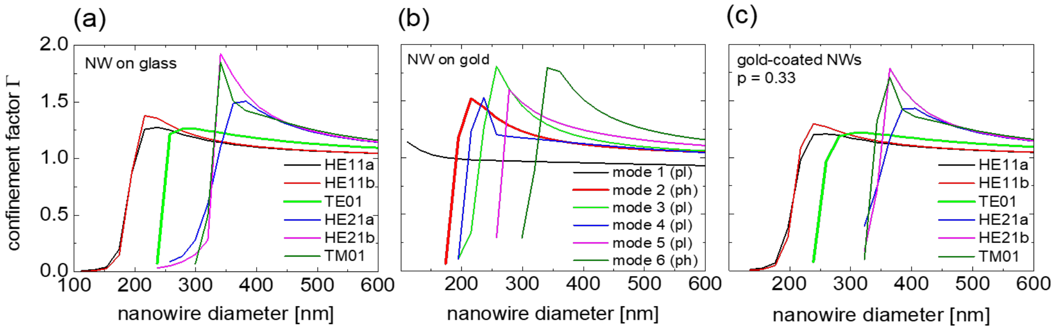

Figure S10: Confinement factor of waveguide modes. **(a)** GaAs NW on glass substrate, **(b)** NW on Au film, and **(c)** Au-coated GaAs NW on glass.

on Au film, the Al_2O_3 coating is therefore not considered as an active A_a. In Au-coated NWs both the Al_2O_3 and the EM Au-coating are excluded from A_a.

The 6 modes supported in the three NW configurations have confinement factors that depend on the NW diameter (Figs. S10 (a), (b), and (c)) The confinement factors of the waveguide modes in bare and in Au-coated NWs on glass are again very similar, showing a rapid decrease near the mode cutoff diameter and a decrease with increasing nanowire diameter.

The confinement factors of GaAs NWs on Au film, shown in Fig. S10 (b), demonstrate a high confinement factor Γ of ~1 for the plasmonic mode 1. In addition, the confinement factor is almost independent of the NW diameter. The confinement factors for modes 2 to 6 decrease swiftly near the mode cut-off diameters and show similar values as in the bare and Au-coated NWs.

Like for the effective refractive indices we approximated the confinement factor Γ of different modes in the conically shaped NW by the average value of the function $\Gamma(d)$.

(d) Facet reflection

The facet reflection of the guided modes in the NWs for the three configurations was calculated

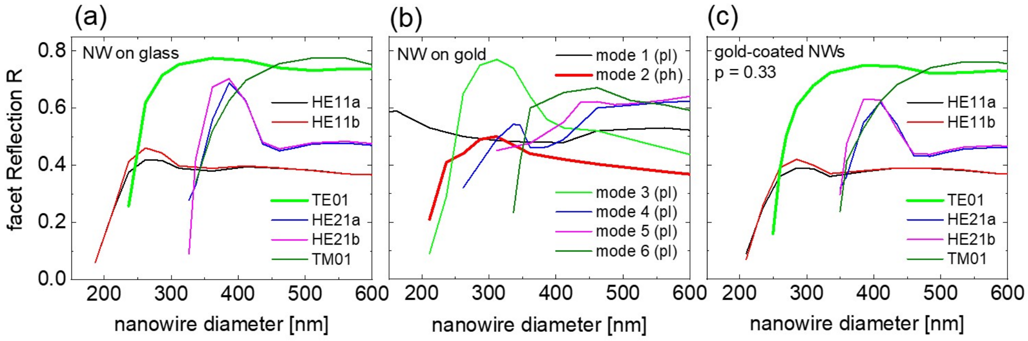

Figure S11: Facet reflection as a function of the nanowire diameter. **(a)** GaAs NW on glass substrate, **(b)** GaAs NW on Au film, and **(c)** Au-coated GaAs NW on glass.

in a similar way as described in the supplemental information in Ref. [1]. Since the broken GaAs NWs are missing the NW tip with the Au catalyst, we only calculated the end facet reflections R_t and R_b at the NW top and base, respectively, at the NW/air interfaces.

The mode reflectivity in a GaAs NW on glass for different guided modes as a function of the nanowire diameter is shown in Fig. S11 (a). When the diameter of the nanowire approaches the threshold diameter, the facet reflection decreases rapidly. The estimated facet reflection in a GaAs NW on Au film as a function of its diameter is presented in Fig. S11 (b). Figure S11 (c) shows the facet reflection in an Au-coated GaAs nanowire on glass as a function of diameter. The facet reflection for all 6 modes shows a similar trend as for the modes in a NW on glass.

Because of the conical shape of the highly Zn-doped NWs the total facet reflection of the nanowires was determined by the geometrical mean of the tip R_t and base facet R_b reflectivity given by $R = \sqrt{R_t R_b}$.

(e) Calculation of the group index n_g

The group index for the waveguide modes was calculated using Lumerical, MODE solutions. The relationship between group index and confinement factor is given by Eq. (S4) [1,11]

$$\Gamma = \frac{n_g}{n_b}\Gamma_0 \quad \text{(S4)}$$

Here, Γ is the confinement factor of the waveguide, n_g is the group index of the waveguide, Γ_0 is the ratio of the energy in the active region of the waveguide to the energy in the whole waveguide, n_b is the effective refractive index of the gain medium [11]. Figures S12 (a), (b), and (c) show the group indices as function of the outer nanowire diameter in the three types of samples. As for the effective refractive indices, we approximated the group indices of different modes in a conically shaped NW by the average value of the functions $n_g(d)$.

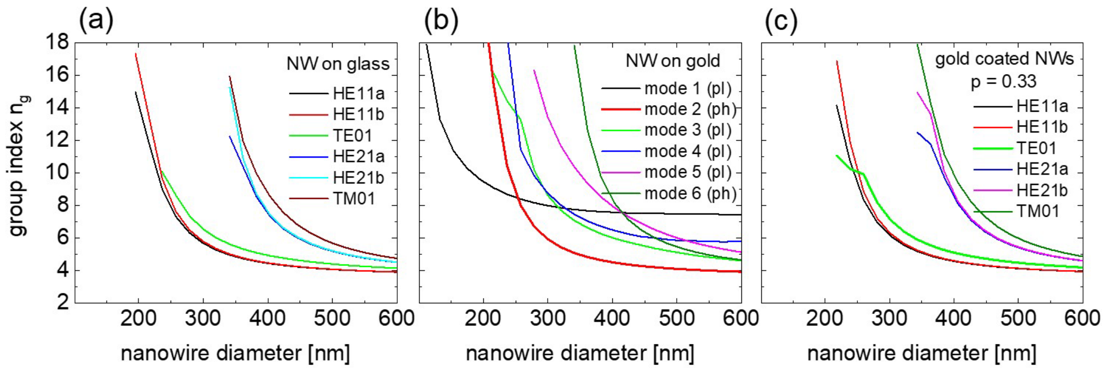

Figure S12: Group index n_g as a function of nanowire diameter. **(a)** GaAs NW on glass substrate, **(b)** GaAs NW on Au film, and **(c)** Au-coated GaAs NW on glass.

Figures S13 (a), (b), and (c) compare the experimentally obtained group index n_g of nanowires with the theoretically calculated averaged n_g values for different modes. This comparison enables the identification of lasing modes in all three design configurations.

Figure 13 (a) shows the calculated group indices of modes HE11a, HE11b and TE01 and the

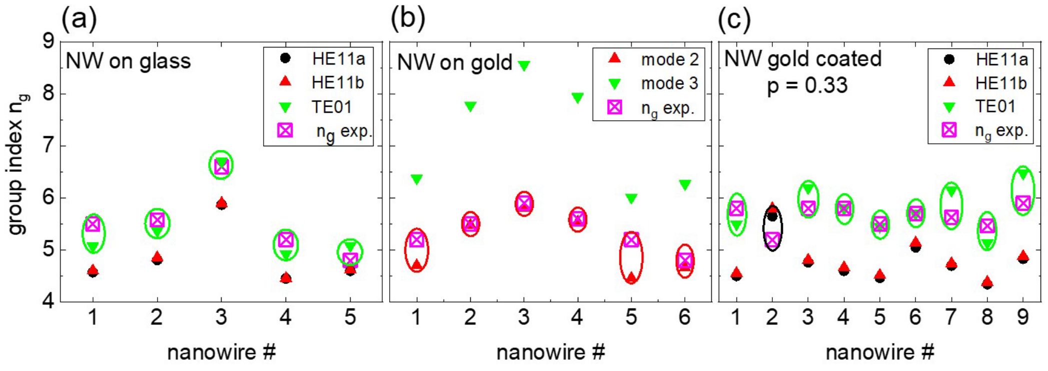

Figure S13: Calculated group index values of various waveguide modes as labelled. **(a)** GaAs NW on glass, **(b)** GaAs NW on Au film and **(c)** Au-coated NW on glass substrate. The experimentally determined group index values are shown as open-crossed symbol.

experimental n_g values of 5 lasing nanowires on glass at 77 K using eq. (S1). The dimensions of the investigated nanowires are given in table ST1. Comparison with calculated values reveals that TE01 mode is the lasing mode in all NWs on glass.

Figure S13 (b) shows the calculated n_g values of GaAs nanowires on Au film for 6 lasing nanowires. The n_g values calculated from Lumerical calculations are in the range of 5 to 6 and correlate well with the experimentally obtained group index of mode 2.

Figure S13 (c) compares the experimentally determined n_g values for all investigated Au-coated nanowires on glass with the theoretically calculated results. Most of the nanowires exhibit n_g values between 5 and 6.5, which is close to the n_g values of the TE01 mode. NW #2 has a group index that is close to the calculated value of HE11a mode because of the small tip diameter of ~230 nm

(corresponding to a core diameter of ~200 nm), which is below the cutoff diameter of the TE01 mode.

(f) Threshold gain calculations

The threshold condition for lasing is calculated using the equation below [9]

$$\Gamma g_{th} = \alpha_p + \frac{1}{L}\ln\left(\frac{1}{R}\right) = \alpha_p + \alpha_R \qquad \text{(S5)}$$

where Γ is the mode confinement factor, g_{th} is the threshold gain of the GaAs NW, L is the length of conical nanowires, $R = \sqrt{R_t \cdot R_b}$ is the mode reflectivity calculated by taking the geometric mean of the reflectivity at the top and bottom end facets. α_p is the plasmonic loss calculated using the imaginary part κ of the effective index of NWs.

All the parameters discussed above are obtained by averaging the parameter functions between the tip and base diameter as explained earlier. With these parameters, the threshold gain values of all supporting modes are calculated and compared to each other. Waveguide modes with the lowest threshold gain are assigned to the observed laser modes. The lowest calculated threshold gain values for the three types of NWs are summarized in tables ST4, ST5 and ST6. The last row of the tables shows calculated parameters and g_{th} values of the "average" model NW in each configuration. The calculated g_{th} values of these "average" model NWs are compared with the threshold gain g_{th} values obtained from the rate equation analysis of lasing nanowires described in section 8.

Table ST4: Parameters to calculate the threshold gain of investigated NWs on glass.

GaAs NW on glass	R	α_R [cm^{-1}]	α_p [cm^{-1}]	Γ	g_{th} [cm^{-1}] [mode]
#1	0.73	720	0	1.18	610 [TE01]
#2	0.59	865	0	1.18	733 [TE01]
#3	0.48	2823	0	1.24	2277 [TE01]
#4	0.74	670	0	1.17	580 [TE01]
#5	0.68	772	0	1.18	650 [TE01]
"Average" NW	0.69	810	0	1.19	680 [TE01]

Table ST5: Parameters to calculate the threshold gain of investigated NWs on Au film.

NW on Au film	R	α_R [cm^{-1}]	α_p [cm^{-1}]	Γ	g_{th} [cm^{-1}] [all mode 2]
# 1	0.45	2575	72	1.13	2340
# 2	0.41	1897	142	1.17	1740
# 3	0.43	2813	167	1.22	2440
# 4	0.44	2932	141	1.20	2560
# 5	0.44	2342	53	1.12	2140
# 6	0.44	2562	68	1.13	2330
"Average" NW	0.44	2414	85	1.15	2175

Table ST6: Parameters to calculate the threshold gain of investigated Au-coated NWs.

Au coated NW on glass	R	α_R [cm^{-1}]	α_p [cm^{-1}]	Γ	g_{th} [cm^{-1}] [mode]
#1	0.72	1368	1019	1.16	2060 [TE01]
# 2	0.33	2080	1325	1.13	3010 [HE11a]
#3	0.68	1260	1293	1.17	2180 [TE01]
#4	0.71	1233	1150	1.17	2030 [TE01]
#5	0.72	1216	1020	1.16	2030 [TE01]
#6	0.63	1170	1570	1.16	2360 [TE01]
#7	0.67	1112	1226	1.16	2020 [TE01]
#8	0.73	925	849	1.15	1540 [TE01]
#9	0.7	1229	1396	1.18	2230 [TE01]
"Average" NW	0.69	1124	1222	1.17	2010 [TE01]

(g) Estimation of the absorption cross-section

The absorption cross-sections σ_{abs} were estimated using a 6-sided truncated pyramid for the Al_2O_3/GaAs NWs on glass, on Au film and for Au-coated NWs. Tapering of the NWs was included by providing the tip and base diameter as well as the length of the truncated nanowire in the Lumerical script. In these calculations, which are important for the rate equation analysis described in section 8, an "average" model NW for each of the three configurations was considered.

The wavelength of the incident light source was λ_p = 720 nm. The light polarization was set parallel and perpendicular to the NW long axis to consider polarization dependent light absorption. The absorption cross-section values of both polarization directions were averaged. In the case of

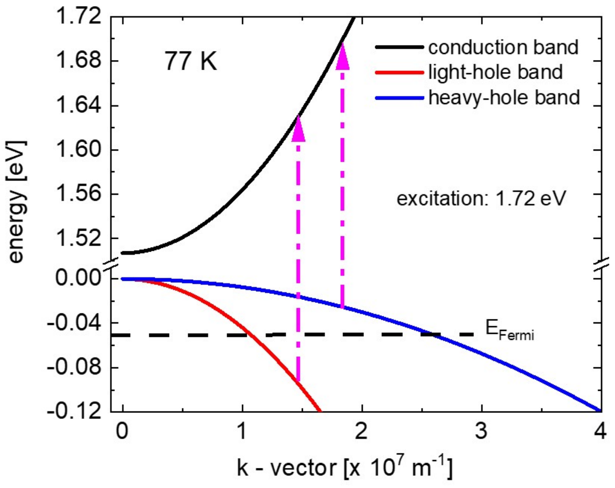

Figure S14: Band-band transitions in the GaAs NW at an excitation energy of 1.72 eV. Due to the position of the Fermi level E_{Fermi} = - 56 meV below the valence band maximum, essentially no optical transition from the heavy-hole band is possible.

the EM Au-coated Al_2O_3/GaAs NW on glass substrate, the absorption cross section in the GaAs core was estimated by subtracting the pump pulse dissipation in the Au-coating. The latter was obtained from an EM Au-coated GaAs NW with an imaginary index value κ set to zero in the GaAs core.

Because of the high doping level of the Zn-doped GaAs NWs ($p = 2\times10^{19}$ cm^{-3}) and the resulting energy position of the Fermi-level E_{Fermi} within the valence band, the optical absorption from the heavy-hole band is essentially blocked. Figure S14 demonstrates this situation at a temperature of T = 77 K where the Fermi energy is calculated to be 56 meV below the valence band minimum. Therefore, for light excitation with an energy of 1.72 eV (λ_p = 720 nm) nearly no electrons are present in the heavy hole-band, while the transition from the light-hole band to the conduction band is unrestricted. Therefore, the calculated absorption cross-section values σ_{abs} were corrected by a blocking factor b_f of approximately b_f = 0.35. At room temperature the blocking of the heavy-hole band to the conduction band is relaxed leading to a correction factor of b_f = 0.6.

Table ST7 summarizes the calculated absorption cross-section values σ_{abs} for the "average" NWs for the different lasing configurations at 77 K. The absorbed pump power η_p in the NW was estimated according to $\eta_p = b_f\ \sigma_{abs} / A_s$, where A_s is the incident laser spot area. For the 295 K experiments the NW dimensions were estimated from the longitudinal mode distances in comparison with Fig. S6 and Fig. 6. The calculated absorption cross-section values σ_{abs} for NWs with estimated dimensions at 295 K are shown in Table ST8.

ST7: Calculated absorption cross-section values for the "average" model NWs in the three configurations at 77 K.

Samples	Average NW length [µm]	Average NW tip diameter [nm]	Average NW base diameter [nm]	Absorption cross-section [cm^2]
NWs on glass	4.6	274	497	$1.14\text{x}10^{-8}$
NWs on Au	3.4	280	460	$1.34\text{x}10^{-8}$
Au coated NWs	3.3	310	499	$5.25\text{x}10^{-9}$

ST8: Calculated absorption cross-section values for the NWs in three configurations at 295 K.

Samples	Estimated NW length [µm]	Estimated NW tip diameter [nm]	Estimated NW base diameter [nm]	Absorption cross-section [cm^2]
NWs on glass	3	350	500	$9.13 \text{ x } 10^{-9}$
NWs on Au	4.2	300	500	$1.83 \text{ x } 10^{-8}$
Au coated NWs	3	350	500	$5.62 \text{ x } 10^{-9}$

4. Determination of NW temperature from photoluminescence spectra

The lattice and the carrier temperature of optically pumped NWs was estimated by comparing the photoluminescence spectra obtained at pump levels below the threshold for amplified spontaneous emission (ASE) with the calculated spontaneous emission spectrum at a specific temperature. The spontaneous emission (luminescence) spectrum of direct, bulk semiconductors is theoretically expressed by [1,12],

$$r_{sp}(\hbar\omega) = \frac{2n_r e^2 \omega}{hc^3 \varepsilon_0 m_0^2} |M|^2 \int \rho_r(E) f_c(E)(1 - f_v(E))\, l(E - \hbar\omega) dE \qquad \text{(S6)}$$

where $\rho_r(E)$ and $f_{c,v}(E)$ are the 3D reduced density of states function and the Fermi-Dirac function for the conduction and valence band, respectively. $l(E-\hbar\omega)=1/\pi\gamma\,\mathrm{sech}\left((E-\hbar\omega)/\gamma\right)$ is the line-shape broadening function which accounts for the energy broadening of electron-hole states. The quantities $e, n_r, \varepsilon_0, c, m_0, \hbar\omega$ and $|M|^2$ are the electron charge, refractive index, vacuum permittivity, vacuum speed of light, electron mass, photon energy and the momentum matrix element. In our calculations, we included contributions from both heavy-hole and light-hole sub-bands. The quasi Fermi-levels $EF_c(n)$ and $EF_V(p+N_A^-)$ at the conduction and at the valence bands of the NWs were calculated using a Polylogarithm function of order 3/2 as described in [13]. *n* and *p* are the photo-excited carrier densities and $N_A^- = 2\times10^{19}$ cm^{-3} corresponds to the density of holes provided by the Zn-acceptors [2]. In the calculation, we used the parameters given in the

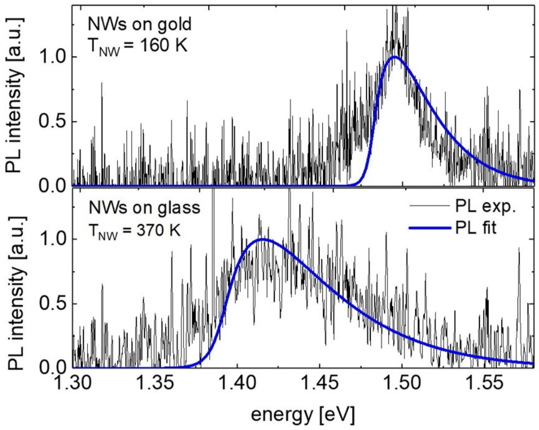

Figure S15: Comparison between experimentally obtained PL spectra from NW on Au film and on glass at T_{cryo} = 77 and 295 K (thin black line) with calculated spontaneous emission curves at a nanowire temperature T_{NW} = 160 and 370 K.

supplementary table 3 of Ref. [2]. We further considered a temperature induced band-gap shrinkage [2].

$$E_g(T)=\left(1.519-5.405\times10^{-4}\frac{T^2}{204+T}\right)\mathrm{eV}\,. \qquad \text{(S7)}$$

The PL spectra obtained from Al_2O_3/GaAs NWs on glass, on Au film and from EM Au-coated NWs at low pump pulse fluences and at a specific cryostat temperature look very similar. Figure S15 shows the PL spectra from NWs on Au film and on glass obtained at cryostat temperatures of T_{cryo} = 77 and 295 K, respectively. The emission band at 1.48 eV at T_{cryo} 77 K is attributed to a Zn-acceptor related transition [2]. The blue full lines show the calculated PL spectra derived with eq. (S6). The calculations reveal that the temperature T_{NW} in the NWs is ~80 to 90 K higher than T_{cryo}. In these calculations, a line-shape broadening γ of 4 and 6 meV was used at both T_{NW} = 160 and 370 K, respectively. The higher NW temperature T_{NW} is attributed to the high rate of non-radiative surface state relaxation.

5. Temperature dependent measurements

A temperature-controlled, continuous nitrogen flow cryostat was used to perform lasing experiments at temperatures ranging from 77 to 295 K. Figure S16 (a) shows temperature dependent lasing spectra from a bare NW on glass at a pump power of 145 mW. The dimensions of the NW obtained from the SEM image shown in Fig, S16 (b) are L = 4.4 µm, tip diameter d_t = 296

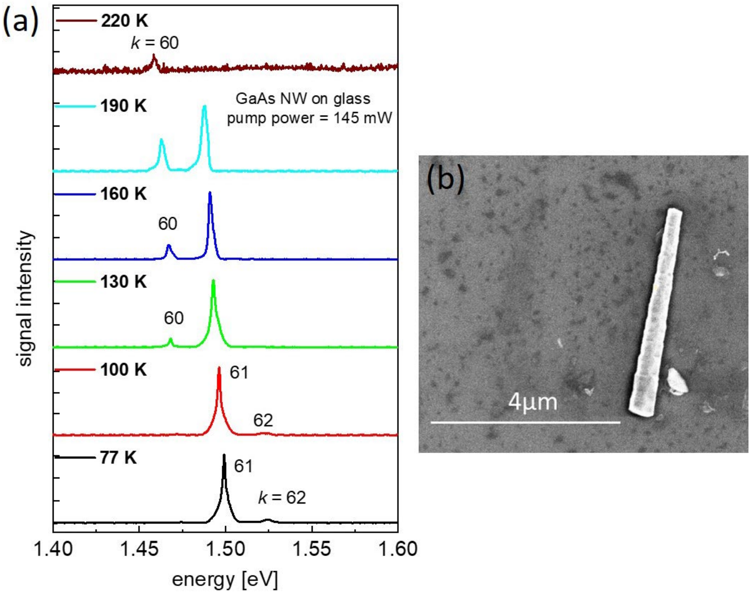

Figure S16: Temperature dependent lasing of NW on glass. **(a)** Lasing spectra as a function of cryostat temperature T_{cryo} obtained from a GaAs NW on glass substrate at a pump power of 145 mW. **(b)** SEM image of the same nanowire.

nm and base diameter d_b = 560 nm. The longitudinal modes have a spectral distance of $\Delta\lambda$ = 13.1 nm resulting in a group index of n_g = 5.74. The red shift of the longitudinal laser lines with mode index k as a function of increasing temperature is caused by a temperature induced change of the effective refractive index. The weakening of the high-energy laser mode k =61 at 1.50 eV is accompanied by an increase of the k = 60 mode at 1.46 eV. This is caused by the temperature-induced shift of the gain spectrum to lower energy, which increases the overlap with lower energy modes at higher temperature.

6. Estimation of the real power emitted from the NWs

In both the lasing spectra and the *L-L* curves the NW laser intensity is given in counts per

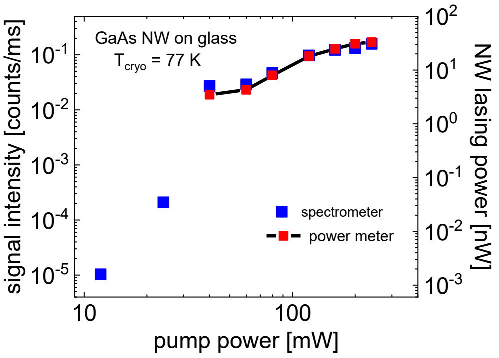

Figure S17: Signal intensity of the NW emission versus pump power (*L-L* plot) obtained from lasing spectra of a GaAs NW on glass (blue squares), and from power meter measurements (red squares) at T_{cryo} = 77 K and λ_p = 720 nm.

millisecond (counts/ms) as acquired by the CCD spectrometer. To estimate the real power emitted from the lasing NW, the detector head of a power meter was placed at the position of the glass fiber

entrance of the spectrometer. The sensitivity of the power meter was ~1 nW. After each pump power increase a measurement with both the spectrometer and with the power meter was performed on the same NW. To measure the power meter background correctly, the NW was moved out of the laser spot of the pump pulse using the micrometer translation stage at the cryostat. The results of these measurements are plotted as the peak power of the NW emission versus pump power (*L-L*) on a logarithmic scale in Fig. S17. The blue symbols represent the signal intensity obtained from the NW. Red symbols show the measured NW laser power at corresponding pump powers. Taking into account a geometrical factor of $A_L/(4\pi f^2)$ = 0.0115 with A_L = 2.54 cm^2 being the cross-sectional area of the microscope objective lens with a focal length of f = 4.2 cm, we estimate that a longitudinal laser line with a peak signal intensity of 0.1 counts per millisecond corresponds to a real emitted NW laser power of approximately 1.7 µW.

7. Modeling of the material gain in GaAs

The gain spectrum as a function of carrier density for direct, bulk semiconductors is theoretically described by [1,12,14]

$$g(\hbar\omega) = \frac{\pi e^2 \omega}{n_r c \varepsilon_0 m_0^2 \omega} |M|^2 \int \rho_r(E)(f_c(E) - f_v(E))\, l(E - \hbar\omega) dE \quad \text{(S8)}$$

where $\rho_r(E)$ and $f_{c,v}(E)$ are the three-dimensional, reduced density of states function and the Fermi-Dirac function for the conduction and valence band, respectively. As described in section 4, $l(E-\hbar\omega) = 1/\pi\gamma\, sech((E-\hbar\omega)/\gamma)$ is the line-shape broadening function. Like in the spontaneous emission calculations the quasi Fermi-levels $EF_c(n)$ and $EF_v(p+N_A^-)$ were calculated using a Polylogarithm function of order 3/2 [13] where n and p are the photo-excited carrier densities and $N_A^- = 2\times10^{19}$ cm^{-3} is the density of holes provided by the Zn-acceptors [2]. The parameters for the calculations are given in the supplementary table 3 of Ref. [2]. We further considered a temperature induced band-gap shrinkage of eq. (S7) as well as a bandgap reduction due to optically excited electron-hole pairs [15,16] given by

$$\Delta E_g(n,T) = -\frac{3.24 * r_s^{-3/4}}{\left[1 + 0.0478 r_s^3 \tau^2\right]^{1/4}} E_{XB} \quad \text{(S9)}$$

In eq. (S9) $E_{XB} = 4.8$ meV is the calculated exciton binding energy (with parameters from [2]) and

$r_s = \frac{1}{a_{XB}}\left(\frac{3}{4\pi n_p}\right)^{1/3}$ is the mean

distance between electron hole pairs with respect to the exciton Bohr radius of a_{XB} = 11.6 nm. n_p is the electron hole-pair density (which is equal to $n = p$) and $\tau = kT/E$ is the lattice temperature normalized with respect to the exciton binding energy.

Figures S18 (a) and (b) show the calculated gain spectra at different optically excited electron-hole pair concentrations, as labelled, and at NW temperatures T_{NW} = 160 and 380 K (which corresponds to T_{cryo} = 77 and 295 K as explained above). In the calculations, we neglected temperature dependent changes of the ionized acceptor density N_A^-, which is a good approximation for degenerately doped semiconductors [17]. As expected, the gain spectrum becomes wider with increasing photoexcited carrier density and the peak gain at the corresponding carrier density shifts

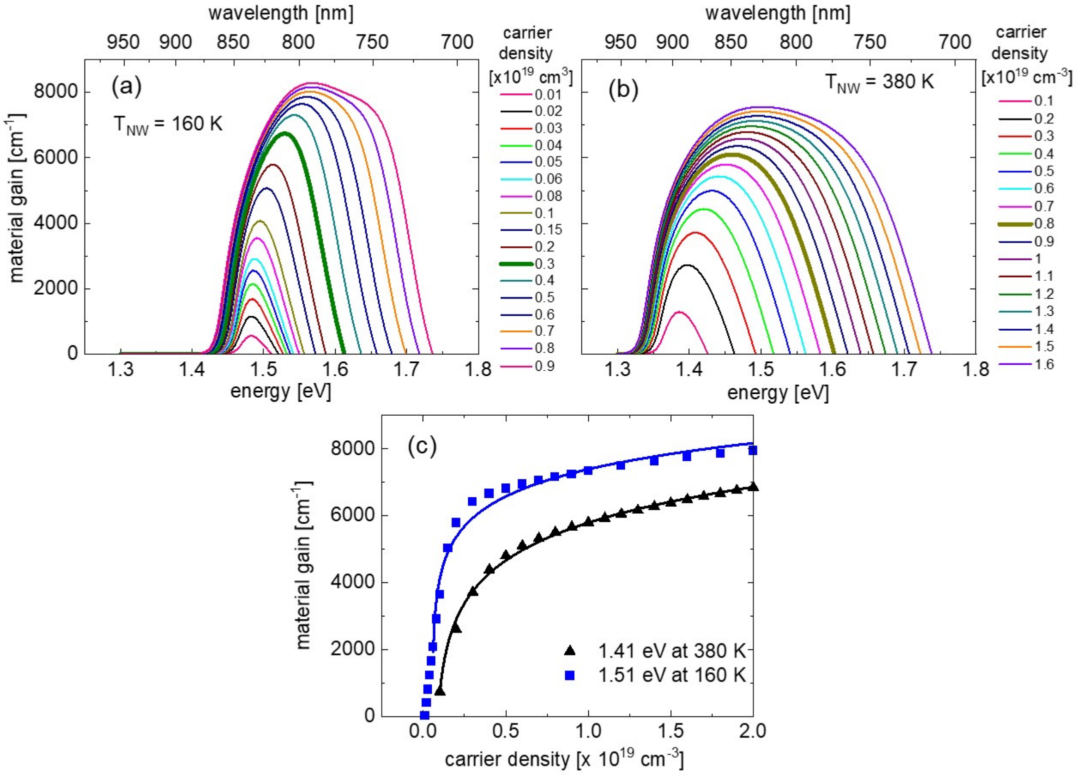

Figure S18: Calculated gain spectra at different photo-excited carrier concentrations as labelled. **(a)** at NW temperatures of 160 K and **(b)** at 380 K. The thick lines indicate the saturation carrier density N_{sat} at which the energy difference between the quasi-Fermi levels is approximately equal to the energy of the exciting laser wavelength of 720 nm. **(c)** Extracted $g(N)$ values (black and blue symbols) at NW lasing energies as labelled. The lines show fits using eq. (S11).

to lower energy with increasing temperature because of the band-gap shrinkage. The thick solid line in the gain spectra indicates the critical carrier density $n = N_{sat}$, where the energy difference between the quasi-Fermi levels $EF(n) - EF(p+N)$ is approximately equal to the energy of the exciting laser wavelength of 720 nm (~1.72 eV). At this point the GaAs NWs are starting to become transparent. Thus, the generation of photoexcited carriers is reduced. We consider this saturation effect when we calculate the NW emission power versus incident pump power (*L*-*L* plot), as described in the next section.

8. Rate equation analysis of NW lasing

In order to analyze the observed lasing behavior of the different NW configurations more quantitatively, we modeled the laser power output versus pump power using a coupled rate equation model for the photo-generated carrier density *N* and for the emitted cavity photons *S,* as described in Refs. [1,12].

$$\frac{dN}{dt} = \frac{\eta_P P(t)}{\hbar\omega V} - AN - BN(N+N_A) - CN(N+N_A)^2 + \sum_i v_g g(N) S_i \, , \tag{S10}$$

$$\frac{dS_i}{dt} = \Gamma v_g (g(N) - g_{th}) S_i + \Gamma \beta B N (N + N_A) \, . \tag{S11}$$

In eq. (S10), the first term on the right side describes the generation rate of electron-hole pairs *N*

R_{rep} = 80 MHz. Quantities $\eta_p, \hbar\omega$ and V are the fraction of pump power that is absorbed, the excited photon energy and the volume of the nanowire or its active region, respectively. η_p is given by $\eta_p = b_f \, \sigma_{abs} / A_s$, where σ_{abs} is the absorption cross section of a nanowire with a particular diameter and length. A_s is the pump pulse spot area and b_f is a blocking factor defined in section S3 (g). σ_{abs} was calculated with FDTD simulations for a laser spot diameter of ~40 µm. To account for the saturation of photo-excited carriers at $\lambda_{p.}$ = 720 nm, η_p was multiplied with a saturation factor ($1-N/N_{sat}$). The saturation density N_{sat} was estimated by the material gain spectra calculated at different temperatures (see Fig. S18). The NW volume was calculated by $V = \pi r^2 L$ with r being the outer radius of the "average" NW of the conically shaped GaAs NW in each configuration and with L being the length of the "average" NW. The second term in eq. (S10) describes the non-radiative recombination of excited carriers, where $A = 2v_S / r$ with the surface recombination velocity v_S. In our calculations, we use the surface recombination velocity of 2.18×10^6 cm s^{-1} as given in Ref. [2].

The third term describes the spontaneous (radiative) recombination rate for highly Zn-doped GaAs NWs. The bimolecular recombination coefficient is $B = (-3.47 \times 10^{-11} \ln N_A + 1.63 \times 10^{-9})$ cm^{3}s^{-1} [18] where N_A is the ionized acceptor density. Auger recombination is considered by the fourth term [12] with a coefficient $C = 3.83 \times 10^{-43} N_A^{0.78}$cm^{6}s^{-1} [19]. The last term in eq. (S10) describes the depletion of carriers by the emission of photons S_i in longitudinal mode i. Here, v_g and $g(N)$ are the group velocity and the gain, respectively. In the calculations we used group index values n_g = 4.7, 5.6, 6.2 for the "average" NWs on Au film, on glass and for Au-coated NWs, respectively.

The first term in the rate equation of emitted photons S in eq. (S11) describes photon generation by stimulated emission into longitudinal mode i. Γ is the mode confinement factor of the "average" NW which has been calculated with Lumerical *MODE solutions* (see section S3 (c)). $g(N)$ is the material gain which can be expressed as

$$g(N) = g_0 \ln\left(\frac{N + N_s}{N_{tr} + N_s}\right) \qquad \text{(S12)}$$

Parameters g_0, N_{tr} and N_s (with N_{tr} being the transparency carrier density and Ns being a shift to keep the logarithm finite at density $N = 0$ [12]) were determined by fitting $g(N)$ with the calculated material gain spectra at the wavelength of typical laser lines.

Table ST9: Parameters g_0, N_s and N_{tr} obtained by fitting the material gain spectra at lasing energies of 1.51 and 1.41 eV at NW temperatures T_{NW} of 160 and 380 K, respectively.

	160 K (at 1.51 eV)	380 K (at 1.41 eV)
g_0 [cm^{-1}]	1083	1460
N_{tr} [x 10^{18} cm^{-3}]	0.549	0.893
N_s [x 10^{18} cm^{-3}]	-0.539	-0.716

Figure S18 (c) shows extracted $g(N)$ values from gain spectra at 160 and 380 K at typical lasing energies of 1.51 and 1.41 eV, respectively. Also shown are fits (solid lines) with parameters g_0, N_s and N_{tr} that are summarized in table ST9. The second term in eq. (S11) considers the generation of photons due to spontaneous emission. The spontaneous emission coupling factor β was adjusted in the calculations to β = 0.15 for NW on glass and to β = 0.2 for Au coated NW and for NW on Au.

λ_0 is the wavelength of the longitudinal mode and n_{eff} and n_g are its effective refractive and group refractive indices.

With the knowledge of $S(t)$, the output power of the nanowire lasers was calculated according to [1,2,12]:

$$P_{out} = \frac{hc}{\lambda_0} \frac{\int_0^T S(t)dt}{T} V_p v_g \alpha_m \qquad \text{(S13)},$$

where T is the inverse of the pump pulse repetition rate, $\alpha_m = \alpha_R + \alpha_p = \Gamma g_{th}$ and V_p is the mode volume V/Γ. The obtained calculated P_{out} was multiplied with an adjustment factor to match the experimental data. In calculating the carrier densities $N(t)$ and the photon density $S(t)$ at different nanowire temperatures (T_{NW} = 160 and 380 K) we neglected any temperature dependence of coefficients A, B, C and D. We also did not consider temperature induced changes of the ionized acceptor density N_A, which are negligible for degenerately doped semiconductors [20].

References

1 Saxena, D. *et al.* Optically pumped room-temperature GaAs nanowire lasers. *Nature Photonics* **7**, 963-968 (2013).

2 Burgess, T. *et al.* Doping-enhanced radiative efficiency enables lasing in unpassivated GaAs nanowires. *Nature Communications* **7**, 11927 (2016).

3 Kaveh, M. *et al.* Exciton emission from hybrid organic and plasmonic polytype InP nanowire heterostructures. *Materials Research Express* **2** (2015).

4 Kaveh, M. *et al.* Controlling the exciton emission of gold coated GaAs-AlGaAs core-shell nanowires with an organic spacer layer. *Nanotechnology* **27**, 485204 (2016).

5 Mohammadi, F. *et al.* Emission dynamics of hybrid plasmonic gold/organic GaN nanorods. *Nanotechnology* **28**, 505710 (2017).

6 Palik, D. *Handbook of Optical Constants*. (Academic Press, 1998).

7 Johnson, P. B. & Christy, R. W. Optical constants of noble-metals. *Physical Review B* **6**, 4370-4379 (1972).

8 Palik, E. D. *Handbook of Optical Constants of Solids*. (Academic Press, 1998).

9 Wickremasinghe, N., Thompson, J., Wang, X., Schmitzer, H. & Wagner, H. P. Controlling guided modes in plasmonic metal/dielectric multilayer waveguides. *Journal of Applied Physics* **117**, 213102 (2015).

10 Cai, W. S., Genov, D. A. & Shalaev, V. M. Superlens based on metal-dielectric composites. *Physical Review B* **72**, 193101 (2005).

11 Ning, C. Z. Semiconductor nanolasers. *Physica Status Solidi B-Basic Solid State Physics* **247**, 774-788 (2010).

12 Coldren, L. A., Corzine, S. W. & Mashanovitch, M. L. *Diode Lasers and Photonic Integrated Circuits*. (Wiley, 2012).

13 Ulrich, M. D., Seng, W. F. & A., B. P. Solutions to the Fermi-Dirac Integrals in Semiconductor Physics using Polylogarithms. *Journal of Computational Electronics* **1**, 431-434 (2002).

14 Chuang, S. L. *Physics of Optoelectronic Devices*. (Wiley, 1995).

15 Zimmermann, R. Nonlinear optics and the mott transition in semiconductors. *Physica Status Solidi B-Basic Research* **146**, 371-384 (1988).

16 Klingshirn, C. Nonlinear optics and dynamics in passive semiconductors. *Festkorperprobleme-Advances in Solid State Phyics* **30**, 335-357 (1990).

17 Stockman, S. A. *et al.* Characterization of heavily carbon-doped gaas grown by metalorganic chemical vapor-deposition and metalorganic molecular-beam epitaxy. *Journal of Applied Physics* **72**, 981-987 (1992).

18 Nelson, R. J. & Sobers, R. G. Minority-carrier lifetime and internal quantum efficiency of surface-free GaAs. *Journal of Applied Physics* **49**, 6103-6108 (1978).

19 Ahrenkiel, R. K., Ellingson, R., Metzger, W., Lubyshev, D. I. & Liu, W. K. Auger recombination in heavily carbon-doped GaAs. *Applied Physics Letters* **78**, 1879-1881 (2001).

20 Bergman, D. J. & Stockman, M. I. Surface plasmon amplification by stimulated emission of radiation: Quantum generation of coherent surface plasmons in nanosystems. *Physical Review Letters* **90**, 027402 (2003).
